# Evolution of the Insecticide Target *Rdl* in African *Anopheles* Is Driven by Interspecific and Interkaryotypic Introgression

**DOI:** 10.1093/molbev/msaa128

**Published:** 2020-05-21

**Authors:** Xavier Grau-Bové, Sean Tomlinson, Andrias O O’Reilly, Nicholas J Harding, Alistair Miles, Dominic Kwiatkowski, Martin J Donnelly, David Weetman, and The Anopheles gambiae 1000 Genomes Consortium

**Affiliations:** m1 Department of Vector Biology, Liverpool School of Tropical Medicine, Liverpool, United Kingdom; m2 Centre for Health Informatics, Computing and Statistics, Lancaster University, Lancaster, United Kingdom; m3 School of Biological and Environmental Sciences, Liverpool John Moores University, Liverpool, United Kingdom; m4 Big Data Institute, University of Oxford, Li Ka Shing Centre for Health Information and Discovery, Oxford, United Kingdom; m5 Wellcome Sanger Institute, Hinxton, United Kingdom; m6 https://www.malariagen.net/projects/ag1000g#people (last accessed June 6, 2020)

**Keywords:** population genomics, insect vectors, insecticide resistance

## Abstract

The evolution of insecticide resistance mechanisms in natural populations of *Anopheles* malaria vectors is a major public health concern across Africa. Using genome sequence data, we study the evolution of resistance mutations in the *resistance to dieldrin locus* (*Rdl*), a GABA receptor targeted by several insecticides, but most notably by the long-discontinued cyclodiene, dieldrin. The two *Rdl* resistance mutations (*296G* and *296S*) spread across West and Central African *Anopheles* via two independent hard selective sweeps that included likely compensatory nearby mutations, and were followed by a rare combination of introgression across species (from *A. gambiae* and *A. arabiensis* to *A. coluzzii*) and across nonconcordant karyotypes of the 2La chromosomal inversion. *Rdl* resistance evolved in the 1950s as the first known adaptation to a large-scale insecticide-based intervention, but the evolutionary lessons from this system highlight contemporary and future dangers for management strategies designed to combat development of resistance in malaria vectors.

## Introduction

The recurrent evolution of insecticide resistance in the highly variable genomes of *Anopheles* mosquitoes (Neafsey et al. 2015; Miles et al. 2017; *Anopheles gambiae* 1000 Genomes Consortium 2019) is a major impediment to the ongoing efforts to control malaria vector populations. Resistance to dieldrin was the first iteration of this cyclical challenge: this organochlorine insecticide was employed in a pioneering vector control program in Nigeria in 1954, but resistant *Anopheles* had already appeared after just 18 months (Elliott and Ramakrishna 1956) due to a single dominant mutation (Davidson 1956; Davidson and Hamon 1962). Dieldrin use ceased in the 1970s due to its high persistence as an organic pollutant and unexpectedly wide toxicity, culminating in a ban by the 2001 Stockholm Convention on Persistent Organic Pollutants. However, resistance has remained strikingly persistent in natural *Anopheles* populations for >40 years (Du et al. 2005). The study of the genetic architecture of dieldrin resistance can thus provide key insights into the evolutionary “afterlife” of resistance mechanisms to legacy insecticides. We address this issue by studying its emergence and dissemination in contemporary African populations of the *A. gambiae* species complex.

Dieldrin resistance in *Anopheles* spp. is caused by mutations in its target site, the γ-aminobutyric acid (GABA) receptor gene, a ligand-gated chloride channel also known as *resistance to dieldrin locus*—or *Rdl—*that is strongly conserved in a wide range of insects (ffrench-Constant, Rocheleau, et al. 1993; Thompson et al. 1993; Du et al. 2005). Two resistance mutations have been found in anophelines, both in codon 296: alanine-to-glycine (*A296G*) and alanine-to-serine (*A296S*)*.* Resistant mutations in the homologous *Rdl* codon have also evolved in other insects, for example, in *Drosophila* spp. (codon 302) (ffrench-Constant, Rocheleau, et al. 1993; Thompson et al. 1993; Du et al. 2005). Populations of *Anopheles gambiae* sensu stricto (henceforth, *A. gambiae*) and its sister species *A. coluzzii* possess both *296G* and *296S* alleles (Du et al. 2005; Lawniczak et al. 2010), whereas the *296S* allele is the only one reported in *A. arabiensis* and the more distantly related malaria vectors *A. funestus* and *A. sinensis* (Du et al. 2005; Wondji et al. 2011; Yang et al. 2017). Normally, dieldrin inhibits the activity of *Rdl* receptors, causing persistent neuronal excitation and rapid death; but codon 296 mutations confer resistance by reducing its sensitivity to the insecticide (ffrench-Constant et al. 2000). However, in the absence of exposure, *Rdl* mutations appear to carry fitness costs, such as lower mosquito mating success (Platt et al. 2015) or impaired response to oviposition and predation-risk signals (Rowland 1991a, 1991b) (although see ffrench-Constant and Bass [2017]). Consequently, with seemingly limited current benefit via exposure to insecticides targeting *Rdl*, persistence of the mutations in anophelines is puzzling.

We interrogate the *Anopheles gambiae* 1000 Genomes cohort (*Anopheles gambiae* 1000 Genomes Consortium 2017, 2019) to ascertain how often dieldrin resistance mutations have evolved in the *A. gambiae*/*A. coluzzii* species pair, and the mechanisms by which these alleles spread across Africa and may persist. We identify two distinct *Rdl* resistance haplotypes in these species, defined by hard selective sweeps and the perfect linkage of the *296G* and *296S* alleles with putatively compensatory mutations. Furthermore, the resistance haplotypes are across genomes from different species (*A. gambiae*, *A. coluzzii*, and *A. arabiensis*), and across chromosomes with differing karyotypes in the 2La inversion (the longest inversion in *Anopheles* genomes) (Coluzzi 2002) within which *Rdl* resides. Interspecies reproductive isolation and inversions such as 2La both result in reduced recombination rates (Sturtevant 1917; Andolfatto et al. 2001; Ayala and Coluzzi 2005; Kirkpatrick 2010), which would in principle hinder the spread of these adaptive alleles. Here, we provide evidence that *Rdl* resistance alleles, which our structural modeling shows have divergent effects on the channel pore, underwent a rare combination of interspecific and interkaryotypic introgression.

Overall, we show that two founding resistance mutations spread with remarkable ease across geographical distance, species, and recombination barriers. This evolutionary trajectory has parallels with later-emerging target-site resistance mechanisms, such as knock-down resistance (*kdr*) mutations in the *Vgsc* gene (Martinez-Torres et al. 1998; Davies et al. 2007; Clarkson et al. 2014, 2018). The persistence of dieldrin resistance also challenges the efficacy of current and newly developed insecticides that also target *Rdl* (Gant et al. 1998; Nakao and Banba 2015; Miglianico et al. 2018), as well as the efficacy of rotative insecticide management strategies (World Health Organization 2012). These results thus emphasize the influence of past interventions on current and future programs of vector population control.

## Results

### Distribution of *Rdl* Resistance Mutations across African Populations

First, we investigated the genetic variation in *Rdl* across populations of the *Anopheles gambiae* species complex, including *A. gambiae* and *A. coluzzii* from the *Anopheles gambiae* 1000 genomes project (*Ag1000G* Phase 2, *n *=* *1,142) (*Anopheles gambiae* 1000 Genomes Consortium 2019), and outgroups from four other species (*A. arabiensis*, *A. quadriannulatus*, *A. melas*, and *A. merus*; *n *=* *36) (Fontaine et al. 2015). All genomes and their populations of origin are listed in supplementary material SM1, Supplementary Material online.

**Fig. 1.**
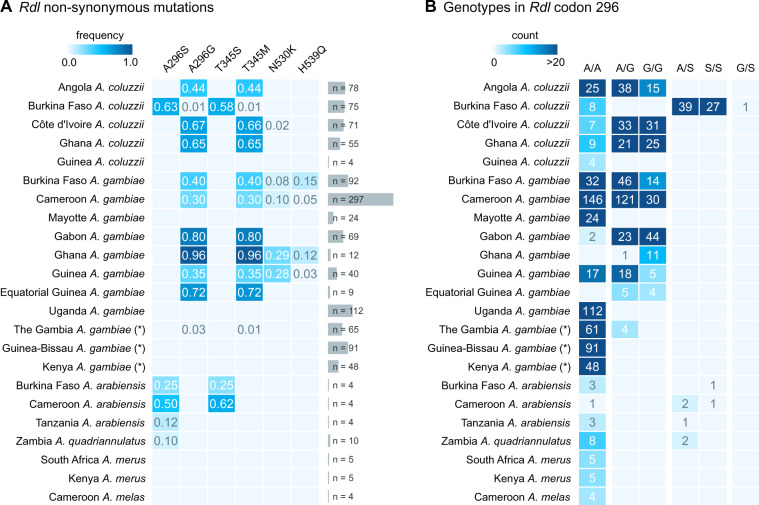
Rdl mutations. (*A*) Frequency of nonsynonymous mutations in *Rdl* across populations of *Anopheles gambiae*, *A. coluzzii* (*Ag1000G* Phase 2), and *A. arabiensis*. Only variants with >5% frequency in at least on population are included. (*B*) Distribution of genotypes for the two mutations in codon 296 (*A296S* and *A296G*). Note: *A. gambiae* populations denoted with an asterisk (The Gambia, Guinea-Bissau, and Kenya) have high frequency of hybridization and/or unclear species identification (see Materials and Methods).

We identified six nonsynonymous mutations that are segregating in at least one population at ≥5% frequency (fig. 1*A*; complete list of variants in supplementary material SM2, Supplementary Material online), including the *296G* and *296S* resistance alleles. *296G* is present in West and Central African populations of both *A. gambiae* and *A. coluzzii*, with frequencies ranging from 30% (Cameroon *A. gambiae*) to 96% (Ghana *A. gambiae*). *296S* is present in *A. coluzzii* specimens from Burkina Faso (63%), as well as *A. arabiensis* (Burkina Faso, Cameroon, Tanzania) and *A. quadriannulatus* (Zambia). Resistance alleles occur as both homozygotes and heterozygotes in all species except *A. quadriannulatus*, which is always heterozygous (fig. 1*B*).

**Fig. 2.**
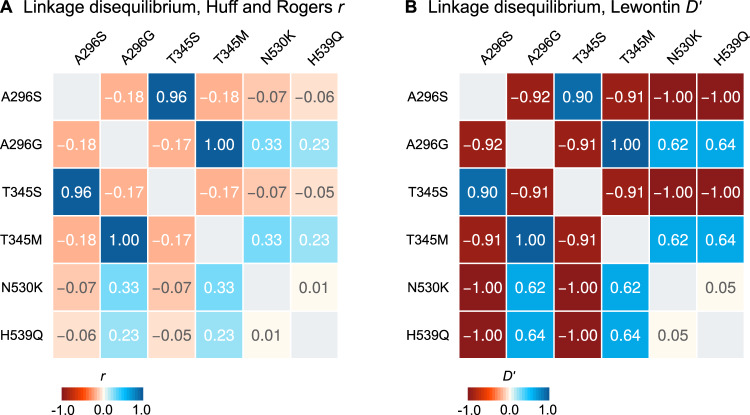
Linkage disequilibrium. Linkage disequilibrium between nonsynonymous mutations in *Rdl*, calculated using Huff and Rogers’ *r* (*A*) and Lewontin’s *D′* (*B*).

We also identified two mutations in codon 345 with very similar frequencies to those of each codon 296 mutation: *T345M* (C-to-T in the second codon position), co-occurring with *A296G*; and *T345S* (A-to-T in the first codon position), co-occurring with *A296S*. The high degree of linkage disequilibrium between genotypes in codons 296 and 345 confirmed that they were co-occurring in the same specimens (fig. 2; e.g., the *296G*/*345M* allele pair had a Huff and Rogers *r* and Lewontin’s *D′* = 1), and was apparent in all individual populations where the alleles were present (supplementary material SM3, Supplementary Material online). Codons 296 and 345 are located in the seventh and eighth exons of *Rdl*, separated by 3,935 bp; and they map to the second and third transmembrane helices of the RDL protein, respectively (supplementary material SM4, Supplementary Material online).

### *Rdl* Resistance Mutations Evolved on Two Unique Haplotypes in *A. gambiae* and *A. coluzzii*

**Fig. 3.**
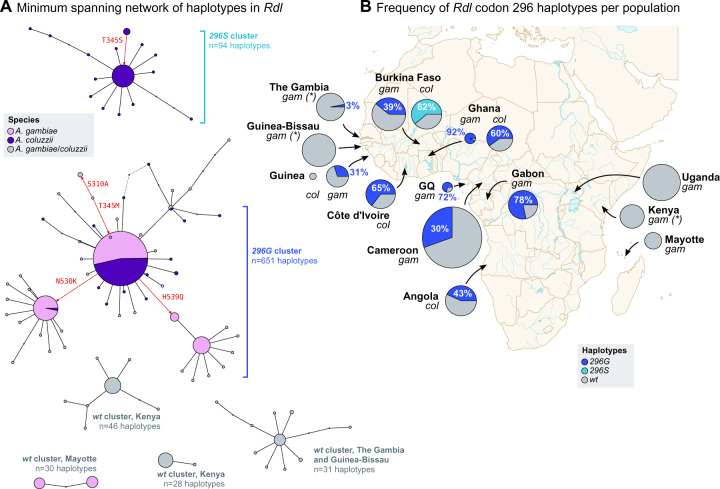
*Rdl* haplotypes. (*A*) Minimum spanning network of haplotypes around *Rdl* codon 296 (626 phased variants located ±10,000 bp from the 2L:25429236 position). Only haplotype clusters with a frequency >1% in the cohort are represented (complete networks available as supplementary material SM6, Supplementary Material online). Each node in the network is color coded according to its species composition. Haplotype clusters carrying the resistance alleles *296G* and *296S* are highlighted in blue. Red arrows indicate the direction of nonsynonymous mutations (relative to reference genome). (*B*) Frequency of resistance haplotypes per population. Pie area reflects sample size, ranging from Guinea *Anopheles coluzzii* (*n* = 8) to Cameroon *A. gambiae* (*n* = 594). Detailed frequencies with absolute counts in supplementary material SM14, Supplementary Material online. *gam, A. gambiae*; *col, A. coluzzii*. *gam* populations denoted with an asterisk have unclear species identification and/or high rates of hybridization.

The high frequency of the *296S* and *296G* alleles in various populations of *A. gambiae* and *A. coluzzii* (fig. 1), together with their co-occurrence with nearby mutations (fig. 2), were suggestive of a selective sweep driven by positive selection on the resistance alleles. To clarify this possibility, we inspected the similarity of haplotypes in *A. gambiae*, *A. coluzzii*, and the four outgroup species (*n *=* *2,356 haplotypes) using a minimum spanning network based on 626 phased variants located 10,000 bp upstream and downstream of codon 296 (fig. 3).

We identified two distinct groups of haplotypes associated with resistance mutations. First, the *296G* cluster contained haplotypes sharing the *296G*/*345M* alleles which were widely distributed in Central and West Africa (11 populations of *A. coluzzii* and *A. gambiae*; *n *=* *651 haplotypes). The *296G* group showed two subclusters associated with the downstream mutations *N530K* and *H539Q* (red arrows in fig. 3*A*), which were present in a subset of mostly *A. gambiae* populations (Guinea, Ghana, Burkina Faso, and Cameroon; fig. 1*A*); with just a few *A. coluzzii* from Côte d’Ivoire in the *N530K* cluster. Both *N530K* and *H539Q* are in partial linkage disequilibrium with *296G* alleles (fig. 2).

In contrast, the *296S* cluster, defined by ubiquitous co-occurrence of the *296S*/*345S* allele pair, was restricted to *A. coluzzii* from Burkina Faso (*n *=* *94; fig. 3*A* and *B*), whereas the *A. arabiensis* and *A. quadriannulatus 296S* haplotypes appeared as distantly related singletons (not visible on fig. 3, see supplementary materials SM5 and SM6, Supplementary Material online). We also found four smaller wild-type clusters (*296A* allele; henceforth *wt*) that are specific to other geographical locations (Kenya, Mayotte, and The Gambia/Guinea-Bissau). The remaining haplotypes are also *wt* and group in smaller clusters or singletons with frequencies <1% in the data set (*n *=* *1,476, 62.6% of all examined haplotypes; supplementary materials SM5 and SM6, Supplementary Material online).

Both the *296G* and *296S* haplotype clusters are often found in high frequencies within their respective populations. For example, *296S* was present in 62.3% of all Burkinabè *A. coluzzii*, and *296G* reached 91.7% in Ghanaian *A. gambiae* (fig. 3*B*).

The haplotype clustering analysis shows that all nonsynonymous mutations (*T345M*, *T345S*, *N530K*, and *H539Q*) are associated with either the *296G* or the *296S* resistance haplotypes. The existence of seven nonsynonymous mutations associated in haplotypes that have evolved over the last 70 years is remarkable: mosquito *Rdl* genes are highly conserved and have accumulated very few amino acid mutations since anophelines diverged from culicines (for instance, *A. gambiae Rdl* retains a 97.6% amino-acidic identity with its *Aedes aegypti* ortholog and *d*_N_*/d*_S_ = 0.052, indicating predominant purifying selection; supplementary material SM4, Supplementary Material online). Here, we observe that the resistant haplotypes accumulate an excess of nonsynonymous mutations compared with the *wt*, with nonsynonymous to synonymous genetic diversity ratios (π_N_*/*π_S_) being ∼18× higher in the *296G* cluster (π_N_*/*π_S_ = 2.428±0.009 SE) than in *wt* haplotypes (π_N_*/*π_S_ = 0.135±0.001); and ∼4× higher in *296S* (π_N_*/*π_S_ = 0.485±0.018).

### The *296S* and *296G* Alleles Are Associated with Hard Selective Sweeps

**Fig. 4.**
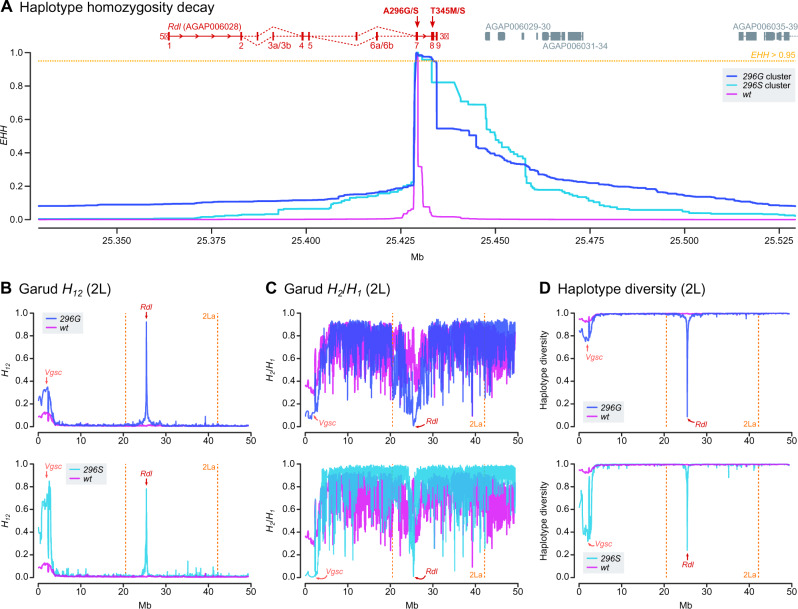
Positive selection of haplotypes carrying resistance mutations. (*A*) Profile of *EHH* decay for each group of haplotypes (*296G*, *296S*, and *wt*), built from 11,180 phased variants located ±100,000 bp from codon 296 (2L:25429236 position). Coordinates of nearby genes are indicated above the *EHH* panel (in *Rdl*, exons are numbered and red arrows indicate the position of codons 296 and 345). (*B–D*) Profiles of Garud *H_12_*, Garud *H_2_*/*H_1_*, and haplotypic diversity along chromosomal arm 2L, highlighting the region covered by the 2La inversion (orange vertical lines) and the location of *Rdl* (red arrow). Each statistic was calculated separately for haplotypes carrying the *296G*, *296S*, and *wt* alleles, using sliding blocks of 500 variants with 20% overlap.

Next, we investigated the signals of positive selection linked to the *296S* and *296G* resistance haplotypes. First, we found that haplotypes carrying *296G* and *296S* alleles had longer regions of high extended haplotype homozygosity (*EHH*) than the *wt* (fig. 4*A*), as expected under a scenario of selective sweeps linked to these resistant variants. A closer examination revealed that *EHH* decays slower at the 3′ region of *Rdl* (fig. 4*A*): in both clusters, *EHH* is >0.95 (i.e., 95% of identical haplotypes) in the region downstream of codon 296 (exons 7 and 8), but decays more rapidly toward the 5′ of the gene (*EHH* < 0.20 in exon 6a/6b, *EHH* < 0.10 in exon 1). The core resistance haplotypes had lengths of 5,344 bp for *296G* and 4,161 bp for *296S* (defined at *EHH* > 95%), which were one order of magnitude higher than *wt* haplotypes (460 bp), and covered all nonsynonymous mutations linked to codon 296 alleles (*T345M*, *T345S*, *N530K*, and *H539Q*).

Next, to estimate the softness/hardness of the sweep, we calculated the profile of Garud’s *H* statistics (Garud et al. 2015) and haplotypic diversity along the 2L chromosome arm (fig. 4*B–D*). Both *296G* and *296S* haplotype clusters showed signals of a hard selective sweep: 1) they had markedly higher Garud’s *H*_12_ (*296G*: 0.698±0.001 SE; *296S*: 0.744±0.006) than *wt* (0.003±0.0), which indicates an overabundance of the most frequent haplotypes in the cohort (Messer and Petrov 2013; Garud et al. 2015); 2) lower *H*_2_*/H*_1_ ratios (*296G*: 0.052±0.0; *296S*: 0.011±0.007) than *wt* (0.756±0.001), indicative of a hard sweep with decreased background variation (Messer and Petrov 2013; Garud et al. 2015); and 3) low haplotypic diversity (*296G*: 0.501±0.001; *296S*: 0.377±0.007) compared with the *wt* (0.998±0.000).

Unexpectedly, chromosomes containing *296G* and *296S* alleles also exhibited signals of positive selection at a distant pericentromeric region of 2L (fig. 4*B–D*), typically associated with strong selective sweeps around the *Vgsc* gene (Lynd et al. 2010; Clarkson et al. 2014, 2018), which is the target site of pyrethroids and DDT (Davies et al. 2007). *Vgsc* selective sweeps are linked to two nonsynonymous substitutions that confer resistance to these insecticides—the *L995F* and *L995S kdr* mutations, commonly known as *L1014F* and *L1014S* after their codon coordinates in *Musca domestica* (Clarkson et al. 2018). Positive selection in *Vgsc* was particularly strong in chromosomes that also carried *296S* alleles (*H*_12_ = 0.917±0.004 SE), followed by *296G* (*H*_12_ = 0.412±0.001) and, to a lesser degree, *wt* (*H*_12_ = 0.147±0.000). However, neither of the *Vgsc kdr* alleles (*995F* and *995S*) is in linkage disequilibrium with *296G* or *296S* (supplementary materials SM7 and SM8, Supplementary Material online). Rather, this apparent association is due to geographical overlap: *296G* and *296S* are present in West African populations that are near-fixed for *Vgsc* resistance alleles (>80% *995F* in 7 out of ten populations; supplementary material SM8, Supplementary Material online), but are mostly absent elsewhere.

Overall, *Rdl* resistance alleles are found on two unique sets of highly similar haplotypes (fig. 3), each of them specific to one allele (*296S* and *296G*), that underwent independent hard selective sweeps (fig. 4).

### Cosegregation of *Rdl* Haplotypes and 2La Inversions

*Rdl* lies within the 2La chromosomal inversion, which is the longest in the *A. gambiae* genome (20.5–42.1 Mb) (Coluzzi 2002). The 2La inversion emerged in the last common ancestor of the *A. gambiae* species complex (Fontaine et al. 2015) and is currently polymorphic in *A. gambiae* and *A. coluzzii* (Stump et al. 2007), where it is linked to a range of important phenotypes including adaptation to human environments (Coluzzi et al. 1979), aridity (Cheng et al. 2012), insecticide resistance (Weetman et al. 2018), and susceptibility to *Plasmodium falciparum* (Riehle et al. 2017). Given that recombination is strongly reduced between chromosomes with discordant inversion karyotypes (Andolfatto et al. 2001; Ayala and Coluzzi 2005; Kirkpatrick 2010), any assessment of the evolution of genes within the 2La inversion, such as *Rdl*, needs to take into consideration whether haplotypes reside in inverted (2La) or noninverted (2L+^a^) backgrounds.

**Fig. 5.**
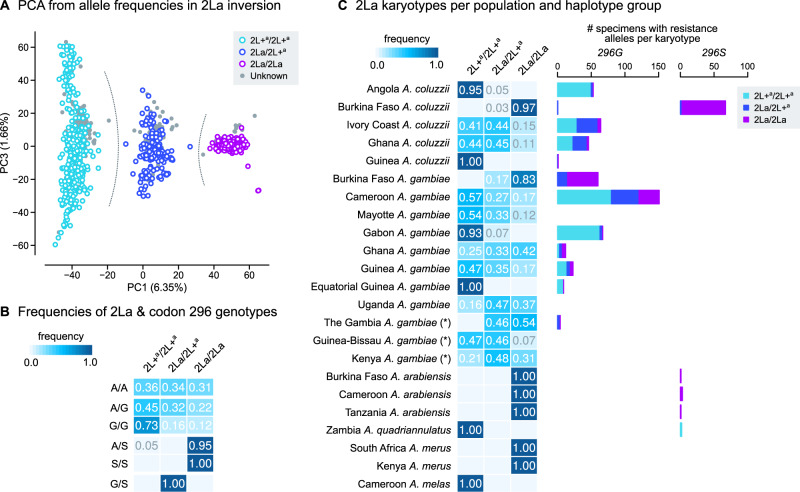
Genotypes of the 2La inversion. (*A*) Principal component analysis of genotype frequencies of 10,000 random variants located within the 2La inversion (coordinates: 2L:20524058–42165532). Specimens from *Ag1000G* Phase 1 are color coded by 2La karyotype (homozygotes and heterozygotes), and they are used as a reference to assign 2La genotypes to Phase 2 specimens (gray). Gray-dotted lines highlight the separation of three clusters according to 2La karyotype. (*B*) Frequency of 2La inversion and *Rdl* codon 296 genotypes. (*C*) Frequency of 2La inversion karyotypes per population (heatmap, left), and number of specimens from each population carrying resistance alleles (*296G* and *296S*), broken down by 2La karyotype (barplots, right). Note: *Anopheles gambiae* populations denoted with an asterisk (The Gambia, Guinea-Bissau, and Kenya) have high frequency of hybridization and/or unclear species identification (see Materials and Methods).

To address this issue, we estimated the 2La inversion karyotypes for the *Ag1000G* Phase 2 samples using a principal component analysis of allele presence/absence in the inverted region (using genomes with known inversion karyotypes as a reference; fig. 5*A* and supplementary materials SM1 and SM9, Supplementary Material online). The first principal component clearly discriminated between each of the inversion genotypes (noninverted 2L+^a^/2L+^a^ homozygotes, inverted 2La/2La homozygotes, and 2La/2L+^a^ heterozygotes). We used this information to compare the frequencies of 2La karyotypes with *Rdl* codon 296 genotypes (fig. 5*B*), and the karyotype frequencies per population (fig. 5*C*). The pan-African *296G* allele is present in all inversion karyotypes, but is more common in noninverted backgrounds (73% of *296G*/*296G* homozygotes have 2L+^a^/2L+^a^ karyotypes; fig. 5*B*), in both *A. gambiae* and *A. coluzzii* populations (fig. 5*C*). On the other hand, *296S* alleles from *A. arabiensis* and Burkinabè *A. coluzzii* occur exclusively within the 2La inversion (100% of *296S/296S* homozygotes are in 2La/2La karyotypes; fig. 5*B*).

### Introgression of *Rdl* Resistance Haplotypes

**Fig. 6.**
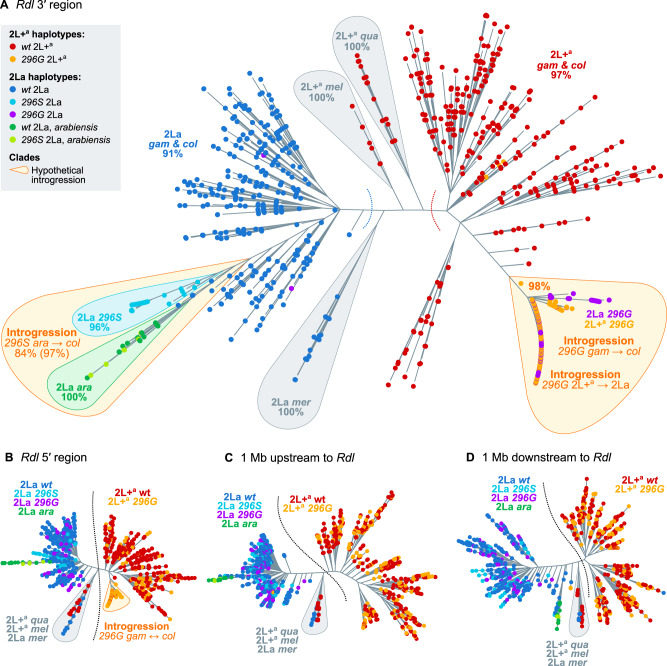
Phylogenies of haplotypes around the *Rdl* locus. (*A*) Maximum-likelihood phylogenetic analysis of variants present at the 3′ region of *Rdl* (20,000 kb). Nodes are haplotypes and have been color coded according to their *Rdl* genotype (*296S*, *296G*, *wt*), 2La karyotype (2La, 2L+^a^), and species. Orange bubbles highlight clades with hypothetical introgression events. Gray bubbles highlight outgroup clades. Statistical supports are shown on selected clades (UF bootstrap). (*B–D*) Analogous phylogenies from the *Rdl* 5′ region, upstream, and downstream regions within the 2La inversion (±1 Mb of *Rdl*). Complete alignments and phylogenies in supplementary materials SM10 and SM11, Supplementary Material online. *col*, *coluzzii; gam*, *gambiae; ara*, *arabiensis; mer*, *merus; mel*, *melas; qua*, *quadriannulatus.* Arrows indicate introgression events.

In order to obtain a more complete picture of possible introgression events, we performed a phylogenetic analysis of haplotype alignments at four loci around *Rdl*: 5′ and 3′ regions of the gene, and two loci upstream and downstream of the gene body (fig. 6). These phylogenies highlight two events of interspecific introgression (explored below in greater detail): *296G* between *A. gambiae* and *A. coluzzii* (as reflected by their identical swept haplotypes; fig. 3), and *296S* between *A. coluzzii* and *A. arabiensis*. In addition, they also confirm the spread of *296G* haplotypes across different 2La inversion types (interkaryotypic introgression; fig. 5). In the following paragraphs, we characterize these introgressions and attempt to identify the donors and acceptors of each event.

#### Interspecific Introgression of *296G* and *296S* Haplotypes

All four phylogenies exhibit two main clades separating *A. gambiae* and *A. coluzzii* haplotypes according to their 2La inversion karyotype, rather than by species (2La in blue, left; 2L+^a^ in red, right; ultrafast bootstrap support [UFBS] 91% and 97%, respectively; fig. 6*A*). This clustering is due to the fact that the 2La inversion has been segregating in *A. gambiae* and *A. coluzzii* since before the beginning of their speciation (Fontaine et al. 2015).

A closer examination shows that *Rdl*-specific phylogenies (fig. 6*A* and *B*) have a distinct subclade within the 2La cluster, consisting of *A. coluzzii 296S* haplotypes and *A. arabiensis*, some of which also possess the *296S* allele (light blue and green sequences in fig. 6*A*; UFBS 97%, 84% for their sister-branch relationship). The deep branching of *A. arabiensis* haplotypes within the *A. gambiae*/*coluzzii* 2La clade is to be expected, as *A. arabiensis* 2La inversions descend from an ancient introgression event from the *A. gambiae*/*coluzzii* ancestor (Fontaine et al. 2015). However, their close phylogenetic relationship with *A. coluzzii 296S* haplotypes is suggestive of interspecific introgression.

**Fig. 7.**
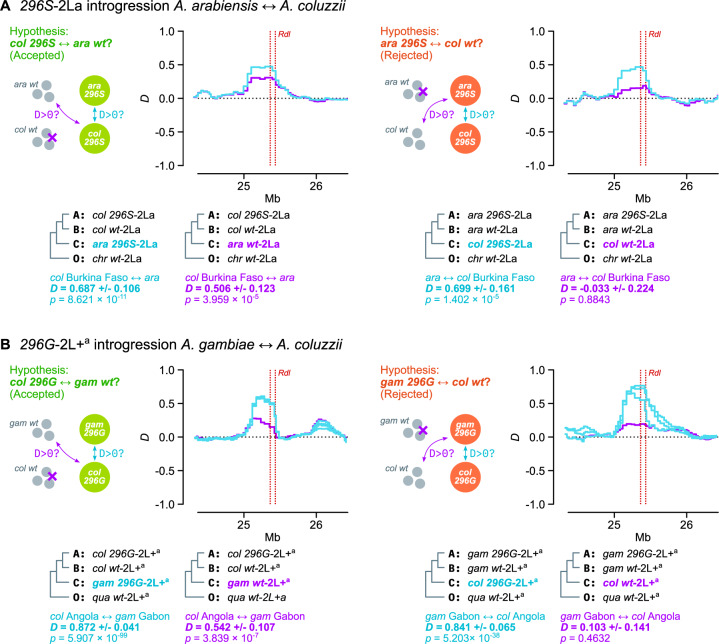
Interspecific introgression. (*A*) Direction of *296S* introgression between *Anopheles arabiensis* and *A. coluzzii* (2La/ 2La background). We test two complementary hypothesis using Patterson’s *D* statistics: left, introgression between *A. coluzzii 296S* homozygotes (population A), *A. coluzzii wt* (B), and *A. arabiensis* (*296S* or *wt*; C) using *A. christyi* as outgroup (O); right, reversing the position of *A. coluzzii* and *A. arabiensis* as populations A/B and C. The complementary hypotheses can be summarized as follows: if *296S* homozygotes from species *i* show evidence of introgression with *wt* homozygotes from species *j* (first test) but not with *wt* from species *i* (second test), *296S* originated in species *j*. (*B*) Direction of *296G* introgression between *A. gambiae* and *A. coluzzii* (2L+^a^/2L+^a^ background), testing two complementary hypothesis using Patterson’s *D* statistics: left, introgression between *A. coluzzii 296G* homozygotes (population A), *A. coluzzii wt* (B), and *A. gambiae* (*296G* or *wt*; C) using *A. quadriannulatus* as outgroup (O); right, reversing the position of *A. coluzzii* and *A. gambiae* as populations A/B and C. Color-coded cladograms at the bottom of each plot indicate the groups of specimens used in each test, including the average *D* in the *Rdl* locus with SEs and *P* values (estimated from the *Z* score of jack-knifed estimates; see Materials and Methods). See detailed lists of comparisons and statistical analyses in supplementary materials SM12 and SM13, Supplementary Material online.

To confirm this event of introgression and ascertain its direction, we compared the results of two complementary Patterson’s *D* tests (fig. 7). The *D* statistic compares allele frequencies between three putatively admixing populations (A, B, and C) and one outgroup (O), and can identify introgression between populations A and C (in which case *D *>* *0) or B and C (*D *<* *0; see Materials and Methods and Durand et al. 2011; Patterson et al. 2012).

Here, if *296S* had emerged in *A. arabiensis* and later introgressed into *A. coluzzii*, we would expect *296S A. coluzzii* specimens to exhibit *D *>* *0 when compared with *296S A. arabiensis*, but also to be more similar to *wt A. arabiensis* (from which *296S* evolved) than to *wt A. coluzzii*. As predicted, we identify evidence of introgression between *A. coluzzii 296S* homozygotes and both 1) *296S A. arabiensis* (*D *=* *0.687±0.106 SE, *P *=* *8.621 × 10^−11^ derived from a *Z*-score distribution) and 2) *wt A. arabiensis* (*D *=* *0.506±0.123, *P *=* *3.959 × 10^−5^; left panel in fig. 7*A*). Conversely, if *296S* had introgressed from *A. coluzzii* into *A. arabiensis*, we would see evidence of introgression between *296S A. arabiensis* and *wt A. coluzzii*, but we do not (right panel in fig. 7*A*;*D* = −0.033±0.224, *P *=* *0.884). These results are robust to various choices of outgroup species (*A. christyi* and *A. epiroticus*), and tests involving a negative control with fixed 2La inversions (*A. merus*) do not show evidence of introgression with *296S* specimens (supplementary material SM12, Supplementary Material online). Thus, we conclude that the *296S* allele originated in *A. arabiensis* and later spread into *A. coluzzii*.

*Rdl* phylogenies (fig. 6*A* and *B*) also show a subclade of highly similar *A. gambiae* and *A. coluzzii* haplotypes within the 2L+^a^ cluster, all of them carrying *296G* alleles. This clade corresponds to the swept haplotypes identified above (fig. 3). We established the polarity of introgression using complementary Patterson’s *D* tests. Here, we found that *296G* haplotypes from resistant *A. coluzzii* populations (Côte d’Ivoire, Angola, and Ghana) exhibited signals of introgression with *wt A. gambiae* from Gabon (e.g., *D *=* *0.542±0.107, *P *=* *3.839 × 10^−7^ compared with Angolan *A. coluzzii*; fig. 7*B*); but that this signal of introgression disappeared when comparing *wt A. coluzzii* to *296G A. gambiae* from Gabon (e.g., *D *=* *0.103±0.141, *P *=* *0.4632 compared with Angolan *A. coluzzii*; fig. 7*B*) or elsewhere (supplementary material SM13, Supplementary Material online). These results support the introgression of *296G* from *A. gambiae* to *A. coluzzii*.

The fact that only Gabonese *A. gambiae* have significant support as the *296G* donor population could indicate that they are closer to the founding *296G* haplotype and/or the original introgression event. However, the negative results in other populations harboring *296G* alleles (Cameroon, Guinea; supplementary material SM13, Supplementary Material online) could also be due to methodological limitations of our analysis—for example, our conservative approach is restricted to specimens that are homozygous for both the inversion karyotype (2L+^a^/2L+^a^) and codon 296 (*296G*/*296G* or *wt*/*wt*); and the similarity between *wt A. gambiae* and *A. coluzzii* relative to the highly divergent swept haplotype can hinder the identification of the original background.

#### The *296G* Haplotype Spread from 2L+^a^ to 2La Chromosomes

The haplotype phylogeny from the *Rdl* 3′ region, where codon 296 variants reside, also revealed that the 2L+^a^ clade (noninverted, red; fig. 6*A*) contained a subcluster of *296G* haplotypes from both 2L+^a^ (orange) and 2La orientations (purple; fig. 6*A*; UFBS 98%). The deep branching of *296G*-2La haplotypes within the 2L+^a^ clade implies that *296G* originated in a noninverted background and later spread to inverted chromosomes via interkaryotypic introgression. Chromosomal inversions are strong barriers to recombination, but double cross-overs or gene conversion events can result in allelic exchange between nonconcordant inversions (Andolfatto et al. 2001; Kirkpatrick 2010) and thus explain this phylogenetic arrangement.

**Fig. 8.**
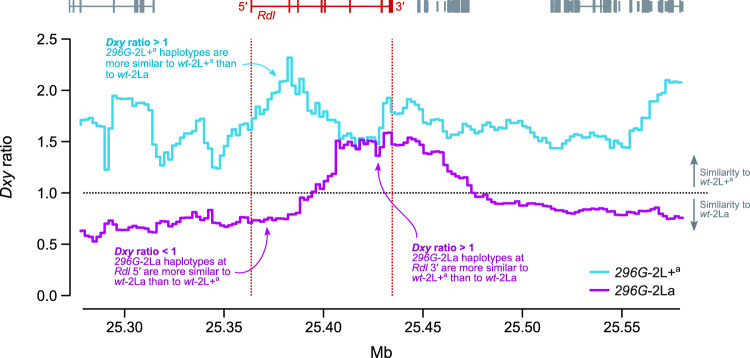
Interkaryotypic introgression of *296G* haplotypes. Ratio of sequence divergence (*Dxy*) between *296G* and *wt* haplotypes of 2L+^a^ and 2La origin. In this ratio, numerators are divergences between *296G* haplotypes (of either 2L+^a^ or 2La origin, in blue and purple respectively) relative to *wt-*2La haplotypes, and denominators are relative to *wt*-2L+^a^. Ratios >1 indicate similarity to *wt*-2L+^a^, and values <1 indicate similarity to *wt-2La.* All values are calculated in windows of 20,000 kp with 10% overlap.

However, the phylogeny of *Rdl* 5′ haplotypes (which excludes codon 296 and the adjacent nonsynonymous mutations) showed that *296G-*2La sequences (purple) branched within the *wt*-2La clade instead (blue; fig. 6*B*). Thus, interkaryotypic introgression only affects the swept haplotype at the 3′ end of *Rdl* (figs. 3 and 4), whereas the 5′ region is closer to the *wt*. We can confirm whether the introgression is specific to the 3′ swept haplotype by examining the profile of sequence divergence along the *Rdl* gene locus (*Dxy;*fig. 8). We expect *296G* haplotypes to be more similar to *wt*-2L+^a^ than to *wt-*2La, given that the *296G* allele first evolved in a 2L+^a^ background (blue line, *Dxy* ratio > 1; fig. 8). In the case of *296G* alleles from 2La chromosomes, this expectation holds at the 3′ region of *Rdl* but not at 5′ nor outside of the gene, where allele frequencies are more similar to the *wt-*2La (purple line, *Dxy* ratio < 1; fig. 8).

The presence of alleles from different karyotypic backgrounds in the *296G*-2La *Rdl* sequences is consistent with the sudden decay of haplotype homozygosity immediately upstream to codon 296 (fig. 4*A*), as the presence of *wt* alleles of 2La origin at 5′ of the *296G* swept haplotypes causes a faster decay in haplotype homozygosity in 2La than in 2L+^a^ haplotypes (supplementary material SM14*A*, Supplementary Material online). Concordantly, haplotype diversity at the 5′ region of *Rdl* is higher in *296G*-2La than in *296G*-2L+^a^ haplotypes (supplementary material SM14*B*, Supplementary Material online).

### Structural Modeling Predicts That *296G* and *296S* Disrupt the Dieldrin Binding Site in Alternative Ways

**Fig. 9.**
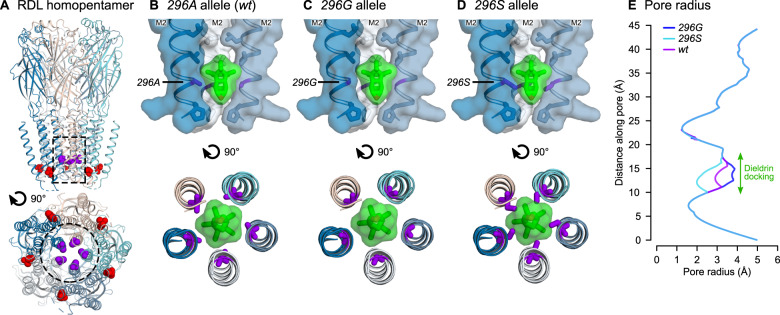
RDL receptor models with docked dieldrin. (*A*) Homology model of the *Anopheles gambiae* RDL homopentamer, viewed from the membrane plane (top) and cytoplasm (bottom). The *296A* (purple) and *345T* (red) positions are shown in space-fill. The dotted outlines depict the receptor regions in panels (*B–D*). (*B*) Docking prediction for dieldrin in the pore of the *296A* (*wt*) receptor. Dieldrin is shown in green, in sticks, and transparent surface. Side chains lining the pore are shown as sticks and *296A* is colored purple. (*C* and *D*) Superimposition of dieldrin docking onto models of the *296G* and *296S* receptors, respectively. (*E*) Pore radii in *296A*, *296G*, and *296S* models.

Finally, we investigated the effects of *296G* and *296S* resistance alleles on the structure of RDL receptors. The *A. gambiae* RDL receptor was modeled as a homopentamer based on the human GABA_A_ receptor structure (Masiulis et al. 2019) (fig. 9). In *wt* receptors, the *296A* residue is located near the cytoplasmic end of the pore-lining second transmembrane helix (M2) and its side chain is orientated into the pore (fig. 9*A*). Residue 345 is located distant from the pore, at the cytoplasmic end of the M3 helix with its side chain orientated toward the lipid bilayer. We carried out automated ligand docking for dieldrin in the *wt* receptor, finding a putative binding site along the receptor pore where the insecticide docked with estimated free energy of binding (*ΔG*_b_) of −8.7 kcal/mol (fig. 9*B*). The *296A* side chains form a major point of contact with the ligand. A structure of human GABA_A_ in complex with picrotoxin showed that this ligand forms multiple hydrogen bonds with residues lining the pore (Masiulis et al. 2019), but dieldrin lacks equivalent hydrogen bond-forming groups. Thus, the close contacts between *296A* side chains and dieldrin suggest that van der Waals interactions between these molecules are the predominant binding interaction.

Next, we superimposed the *wt* dieldrin docking coordinates onto models of resistant RDL receptors, resulting in disruptions of the predicted form of interaction (fig. 9*C* and *D*). The *A296G* substitution widens the pore at the dieldrin docking site (2.9–3.8Å) and reduces the surface area of contact between the lumen and dieldrin (fig. 9*C* and *E*). *A296S* has the opposite effect: it results in a narrower pore (2Å) and shows an overlap between the serine side-chains and dieldrin, which indicates that steric hindrance could prevent the insecticide from binding at this location (fig. 9*D* and *E*).

## Discussion

### Evolution of *Rdl* Resistance: Selective Sweeps and Multiple Introgression Events

Contemporary dieldrin-resistant *A. gambiae* and *A. coluzzii* appear to descend from two unique hard selective sweeps linked to the *A296G* and *A296S* mutations, respectively (figs. 3 and 4). Both sweeps occurred independently on different genomic backgrounds (fig. 6), and have undergone at least three introgression events (figs. 6–8): 1) *296G* from *A. gambiae* to *A. coluzzii*; 2) *296G* from 2L+^a^ to 2La chromosomes; and 3) *296S* from *A. arabiensis* to *A. coluzzii*.

In the case of *296G*, our data support an origin in *A. gambiae* with 2L+^a^ chromosomes, followed by interspecific introgression into *A. coluzzii*, and interkaryotypic introgression into 2La chromosomes. The *A. gambiae* origin is inferred from the background similarity between *A. coluzzii* swept haplotypes and *A. gambiae wt* specimens from Gabon (according to Patterson’s *D* test; fig. 7*B*). *Anopheles gambiae* resistance haplotypes have accrued more nonsynonymous mutations than *A. coluzzii* (*N530K* and *H539Q*; fig. 1*A*), which is consistent with a longer evolutionary history in the former. In either case, the swept haplotype currently spans populations of both species across West and Central Africa—mimicking the pan-African selective sweep described for the homologous *Rdl* mutation in *D. melanogaster* (ffrench-Constant, Rocheleau, et al. 1993; ffrench-Constant, Steichen, et al. 1993; Thompson et al. 1993). This result is in line with previous studies that had hypothesized the existence of a pan-African *296G* sweep due to the strong genetic differentiation found in this locus (Lawniczak et al. 2010).

The interkaryotypic introgression of *296G* haplotypes from noninverted 2L+^a^ into 2La chromosomes (figs. 6 and 7) also facilitated the spread of *296G* resistance alleles, for example, in *A. gambiae* populations with high frequencies of 2La/2La karyotypes such as Burkina Faso (fig. 5*C*). This introgression event affected a short region around codon 296 at the 3′ end of *Rdl*, which contributes to the faster decay in haplotype homozygosity immediately upstream to the resistance mutations (fig. 4*A* and supplementary material SM14*A*, Supplementary Material online). Although it is generally acknowledged that chromosomal inversions strongly suppress recombination (Sturtevant 1917), genetic exchange can occur via double cross-over recombination or gene conversion (Chovnick 1973; Rozas and Aguadé 1994; Andolfatto et al. 2001; Kirkpatrick 2010). The reduction in recombination is weaker in regions distant from the inversion breakpoints (Andolfatto et al. 2001), as it is the case for *Rdl* (located ∼4.8 and ∼16.7 Mb away from the 2La breakpoints), which results in reduced differentiation at the center of the inversion (Stump et al. 2007; Cheng et al. 2012) (supplementary material SM15, Supplementary Material online). To the best of our knowledge, reports of adaptive introgression of individual genes within inversions are rare. In *Anopheles*, one of such cases are certain loci involved in adaptation to desiccation, which are linked to 2La inversions but are exchanged in 2La/2L+^a^ heterozygotes (Cheng et al. 2012; Ayala et al. 2019). Another example, possibly linked to gene conversion, could be the *APL1* cluster of hypervariable immune genes: their pattern of sequence variation is more strongly influenced by geography and species (*A. gambiae*/*A. coluzzii*) than by the 2La inversion where they reside (Rottschaefer et al. 2011).

On the other hand, the *296S* selective sweep has a more restricted geographical distribution. In the *Ag1000G* cohort, *296S* is only found in *A. coluzzii* from Burkina Faso (fig. 3). We also identify *296S* alleles in *A. arabiensis* specimens from East (Tanzania), Central (Cameroon) and West Africa (Burkina Faso); as well as two *A. quadriannulatus* specimens from Zambia (which appears to be the first record in this species; fig. 1*B*).

Interestingly, we find clear evidence of *296S* introgression from *A. arabiensis* into *A. coluzzii* even when comparing with *A. arabiensis wt* specimens (fig. 7*A*), and despite the fact that none of the *A. arabiensis 296S* share the *A. coluzzii* swept haplotype (figs. 3*A* and 6*A*; supplementary material SM6, Supplementary Material online). Thus, lack of genomic evidence from *A. arabiensis* precludes the identification of the actual donor haplotype. A wider sampling of *A. arabiensis* populations will be necessary to complete the picture of *296S* evolution, in order to 1) identify the number of historical *A296S* mutations in this species; 2) establish whether they were associated with one or more selective sweeps; and 3) whether any of these hypothetical sweeps introgressed into *A. coluzzii*.

### Persistence of *Rdl* Mutations after Dieldrin Withdrawal

*Rdl* is a highly conserved gene, with an extreme paucity of nonsynonymous mutations over >100 Ma of evolutionary divergence (Neafsey et al. 2015) in culicines and anophelines, and low *d*_N_*/d*_S_ ratios that indicate a prevalence of purifying selection (supplementary material SM4, Supplementary Material online). In this context, the persistence of *296G* and *296S* alleles in natural populations for >70 years, in spite of its fitness costs in the absence of insecticide (Rowland 1991a, 1991b; Platt et al. 2015), has been a long-standing puzzle.

Our study provides two key insights to this question. First, we find that, relative to the *wt*, haplotypes with resistance alleles have an excess of nonsynonymous genetic diversity (∼18× increase in π_N_*/*π_S_ in *296G*, ∼4× in *296S*). This observation suggests that the emergence of *296G* and, to a lesser degree, *296S*, has substantially altered the selective regime of *Rdl* and enabled the accumulation of additional nonsynonymous mutations in an otherwise highly constrained protein. This accelerated rate of protein evolution appears to have occurred in the only copy of the resistance haplotype, as there is no evidence of copy number variation polymorphisms affecting *Rdl* in the *Ag1000G* data set (Lucas et al. 2019; *Anopheles gambiae* 1000 Genomes Consortium 2019). A similar change has been recently observed for resistance mutations in *Vgsc* (the target site of pyrethroids), whereby *995F* haplotypes accumulate an excess of amino-acidic substitutions (Clarkson et al. 2018).

Second, we identify a high degree of genetic linkage between the *296G*/*345M* and *296S*/*345S* allele pairs, which is observed in all West African populations where codon 296 mutations are present (figs. 1 and 2;supplementary material SM3, Supplementary Material online) due to the fact that virtually all swept haplotypes include both mutations (figs. 3 and 4). This near-universal association is highly relevant because codon 345 mutations are suspected to have compensatory effects that offset the costs of codon 296 variants (Remnant et al. 2014; Taylor-Wells et al. 2015). Studies of fipronil resistance have shown that both the *296G* allele and the combination of *296G* and *345M* alleles resulted in decreased insecticide sensitivity in *A. gambiae* (Taylor-Wells et al. 2015), *D. melanogaster* (Remnant et al. 2014), and *D. simulans* (Le Goff et al. 2005). Crucially, Taylor-Wells *et al.* (2015) showed that, in addition to fipronil resistance, the *A. gambiae 296G* allele causes heightened sensitivity to the GABA neurotransmitter (possibly contributing to the observed fitness costs; Rowland 1991a, 1991b; Platt et al. 2015); and that the addition of the *345M* mutation reduces these detrimental effects while still conferring resistance.

Interestingly, our structural modeling analyses predict opposite resistance mechanisms for each resistance allele: *296G* results in a wider RDL pore with reduced van der Waals interactions with dieldrin (fig. 9*C* and *E*); whereas *296S* narrows the pore and impedes dieldrin docking due to steric hindrance (fig. 9*D* and *E*). These two effects suggest the possibility that the mechanisms behind the hypothesized compensatory roles of codon 345 mutations could be different as well, and open a new line of inquiry to investigate the exclusive association of each resistance variant with downstream mutations (*296G* with *345M*, *296S* with *345S*). Yet, the exact nature of the interaction between these codon 296 and 345 mutations remains unclear. Firstly, residue 345 does not have direct contacts with dieldrin or residue 296 (fig. 9*A*), and changes on its side chain do not directly affect the pore conformation. Secondly, indirect effects are uncertain too: in human receptors, mutations at the interface between the third and second transmembrane helices (where residues 345 and 296 reside, respectively) affect the transition to the desensitized functional state (Gielen et al. 2015); but residue 345 in *A. gambiae* is not buried in this interface and is instead facing the lipid bilayer (fig. 9*A*), and the predicted effects of mutations *T345M* and *T345S* are not obvious.

Other possible factors behind the persistence of *Rdl* resistance alleles include the long half-life of dieldrin as an environmental organic pollutant; as well as the fact that it is the target site of insecticides other than dieldrin. The use of fipronil as a pesticide has been proposed to explain the high frequencies of *Rdl* mutations after dieldrin withdrawal from specific sites (Wondji et al. 2011; Kwiatkowska et al. 2013), for example, in *A. coluzzii* from the Vallée du Kou (Burkina Faso) (Kwiatkowska et al. 2013). Neonicotinoids (imidacloprid) and pyrethroids (deltamethrin) could also contribute to *Rdl* mutation maintenance, as they interact with *Rdl* as a secondary target when used at high concentrations (Taylor-Wells et al. 2015). Pyrethroids have been a major vector control tool across most of sub-Saharan Africa (van den Berg et al. 2012; Oxborough 2016) in the years prior to the collection of the samples used in this study (up to 2012). Finally, other drugs known to interact with *Rdl* have a less clear possible connection with the persistence of the *296G* or *296S* alleles. For example, isoxazolines and meta-diamides are still effective in the presence of codon 296 mutations (Ozoe et al. 2010; Nakao et al. 2013; Asahi et al. 2015), which suggests that they are unlikely to be a primary cause of the maintenance of these alleles in natural populations. *Rdl* is also a secondary target of ivermectin (Chaccour et al. 2013). This drug does not bind in proximity to codon 296, but the *296S* allele nevertheless appears to reduce ivermectin interaction with an in vitro-expressed GABA receptors in *Drosophila* (Nakao et al. 2015). Ivermectin was introduced into mass drug administration campaigns in the 1990s, first for onchocerciasis, then lymphatic filariasis (Hoerauf et al. 2011). Although the interaction of ivermectin with *Rdl* resistance alleles in vivo is not currently understood, these mutations have persisted for two decades between the discontinuation of cyclodiene use and the first mass ivermectin administration campaigns.

### Implications for Vector Control

The apparent ease with which *Rdl* adaptive haplotypes have spread across the barriers to recombination posed by species isolation (*A. gambiae*/*A. coluzzii* and *A. arabiensis*/*A. coluzzii*) and nonconcordant chromosomal inversions (2L+^a^/2La) mirrors previous findings in *Vgsc* target-site mutations (Clarkson et al. 2014), and suggests worrying consequences for insecticide deployment programs. Burkina Faso, where resistance alleles have traversed both barriers to recombination, is a case-in-point example of this risk: the high frequency of 2La inversions (fig. 5*C*) did not prevent the spread of *296G*, and interspecific introgression of *296S* from *A. arabiensis* compounded this problem in *A. coluzzii*.

Also noteworthy is the overlap of *Rdl* and *Vgsc* resistance variants in West and Central Africa. The lack of genetic linkage between *Vgsc* and *Rdl* resistance haplotypes suggests that this co-occurrence is purely geographical, and does not fit a hypothetical epistatic relationship (supplementary materials SM7 and SM8, Supplementary Material online). Yet, this overlap is still relevant for vector control: as pyrethroid resistance increases in *Anopheles* populations (Ranson et al. 2011), the search for substitutes should take into account that some can be rendered ineffective by *296S* or *296G* (e.g., fipronil, Gant et al. 1998; ivermectin, Miglianico et al. 2018; or, possibly, neonicotinoids such as imidacloprid; Taylor-Wells et al. 2015). This risk is currently highest in the West and Central African populations of *A. gambiae* and *A. coluzzii* where both *296G* and *Vgsc 995F* (Clarkson et al. 2018) are common (supplementary material SM8, Supplementary Material online). In the future, the introgression of *296S* from East African *A. arabiensis* could further compound current complications caused by the already high frequencies of *Vgsc 995S* in this region (Clarkson et al. 2018).

This case study of the mechanisms that underlie persistence of dieldrin resistance is also relevant for integrated resistance management. Strategies such as insecticide rotations or mosaics rely on a gradual decline in resistance over time (World Health Organization 2012). Instead, *296G* and *296S* haplotypes have accumulated additional nonsynonymous mutations (fig. 3*A*), some of which (codon 345) are putatively compensatory. As mentioned above, a similar altered selective regime has also been observed in *Vgsc* haplotypes with *kdr* mutations (Clarkson et al. 2018). Interestingly, a study of Brazilian *Aedes aegypti* found that *Vgsc kdr* mutations did not decrease in frequency after a decade without public pyrethroid spraying campaigns (Macoris et al. 2018). Brazilian *Aedes* have a longer history of pyrethroid-based treatments than African *Anopheles* spp. (van den Berg et al. 2012; Macoris et al. 2018); thus, their resilient *kdr* mutations could be 1) recapitulating our observations with respect to *Rdl* and dieldrin, and 2) prefiguring a similar persistence of *Vgsc kdr* in the *A. gambiae* complex after a future phasing-out of pyrethroids in response to their decreasing efficacy (Ranson et al. 2011).

Overall, our results show that the *Rdl* resistance mutations that appeared after the pioneering deployment of dieldrin in the 1950s will still be relevant in the immediate future. Continued monitoring is thus necessary to understand the evolving landscape of genomic variation that underlines new and old mechanisms of insecticide resistance.

## Materials and Methods

### Data Collection

We used genome variation data from *A. coluzzii* and *A. gambiae* mosquitoes from the *Anopheles gambiae* 1000 Genomes Phase 2-AR1. This data set consists of 1,142 wild-caught mosquitoes (1,058 females and 84 males) from 33 sampling sites located in 13 sub-Saharan African countries (supplementary material SM1, Supplementary Material online). To ensure population representativeness, the *Anopheles gambiae* 1000 Genomes Consortium aimed at minimum sample size of 30 specimens per country (Miles et al. 2017) and avoided confounding factors during collection (e.g., insecticide resistance). The list of locations includes continental and island populations, and covers different ecosystems (including rainforest, coastal forests, savannah, woodlands, and grasslands; details in Miles et al. 2017; *Anopheles gambiae* 1000 Genomes Consortium 2019). Specimens were collected at different times between 2009 and 2012 (with the exception of samples from Gabon and Equatorial Guinea, collected in 2000 and 2002, respectively).

The methods for genome sequencing and analysis of this data set have been previously described in detail as part of the Phase 1 and Phase 2 releases of *Ag1000G* (Miles et al. 2017; *Anopheles gambiae* 1000 Genomes Consortium 2019). Briefly, DNA was extracted from each of the 1,142 mosquitoes using Qiagen DNeasy blood and tissue kit (Qiagen Science) and sequenced with the Illumina HiSeq 2000 platform (Wellcome Sanger Institute, UK) using paired-end libraries (100-bp reads with insert sizes in the 100–200 bp range) and aiming at a 30× coverage per specimen (see original papers for details). Variant calling was performed using *bwa* 0.6.2 (Li and Durbin 2009) and the *GATK* 2.7.4 *UnifiedGenotyper* module (Van der Auwera et al. 2013). Haplotype phasing was estimated with *SHAPEIT2* (Delaneau et al. 2013), and variant effects were predicted using *SnpEff* 4.1b (Cingolani et al. 2012).

We retrieved the phased genotype calls, SNP effect predictions, and the array of accessible genomic positions for each of the 1,142 specimens from the *Ag1000G* Phase 2-AR1 online archive (*Anopheles gambiae* 1000 Genomes Consortium 2017). We also obtained the same data for populations of four species in the *Anopheles* complex (*A. arabiensis*, *A. quadriannulatus*, *A. melas*, and *A. merus*) and two outgroups (*A. epiroticus* and *A. christyi*) (Neafsey et al. 2015), as available in the *Ag1000G* online archive (*Anopheles gambiae* 1000 Genomes Consortium 2017). The complete list of genomes with accession codes is available in supplementary material SM1, Supplementary Material online.

The reference gene annotation of *A. gambiae* was obtained from Vectorbase (Giraldo-Calderón et al. 2015) (GFF format, version AgamP4.9). Gene and variant coordinates employed in this study are based on the AgamP4 version of the genome assembly.

### Genotype Frequencies and Linkage Disequilibrium

We retrieved all nonsynonymous genomic variants located within the coding region of *Rdl* (genomic coordinates: 2L:25363652–25434556) that were biallelic, phased, and segregating at >5% frequency in at least one population (henceforth, “nonsynonymous variants”). Parsing and filtering of genotype calls from *Ag1000G* was done using the *scikit-allel* 1.2.1 library (Miles and Harding 2017) in Python 3.7.4.

We calculated the linkage disequilibrium between each pair of nonsynonymous variants using 1) Rogers’ and Huff *r* correlation statistic (Rogers and Huff 2009), as implemented in *scikit-allel* (*rogers_huff_r*); and 2) Lewontin’s *D′* statistic (Lewontin 1964), as implemented in Clarkson et al. (2018).

### Haplotype Networks

We constructed a network of haplotype similarity using 626 biallelic, phased, and nonsingleton (shared between more than two samples) variants located in a region ±10 kb of *Rdl* codon 296 (middle nucleotide, coordinate 2L:25429236). We used the presence/absence of each allele within this genomic region to calculate Hamming distances and build minimum spanning networks (Bandelt et al. 1999), using the *hapclust* function from Clarkson et al. (2018) (with distance breaks >3 variants). Network visualizations were produced using the *graphviz* 2.38.0 Python library (Ellson et al.), with haplotype clusters being color-coded according to species, population and presence/absence of the resistance alleles in codon 296 (*296S*, 2L:25429235; *296G*, 2L:25429236) and the 995th codon of *Vgsc* (fig. 3 and supplementary materials SM5 and SM6, Supplementary Material online). The network visualization in figure 3*A* excludes singletons and haplotype clusters with a cohort frequency <1%.

We calculated the sequence diversity (π) of each haplotype group in the same region (*sequence_diversity* function in *scikit-allel*), using a jack-knife procedure (iterative removal of individual haplotypes without replacement) (Tukey 1958) to estimate the average and SE. We also calculated the sequence diversity in nonsynonymous coding variants from this region (π_N_), synonymous coding variants (π_S_), and their ratio (π_N_*/*π_S_).

### Positive Selection in Haplotype Clusters

We analyzed the signals of positive selection in three haplotype groups, divided according to alleles in codon 296: *wt* (*n *=* *1,476), *296S* (*n *=* *94), and *296G* (*n *=* *651) (supplementary material SM5, Supplementary Material online). First, we calculated the *EHH* decay of each group of haplotypes, using 22,910 variants (phased and biallelic) located ±200 kb of codon 296 (2L:25429236) (using the *ehh_decay* utility in *scikit-allel*). For each haplotype group, we recorded the genomic region where *EHH* decay >0.95 and <0.05.

Second, we calculated the profile of Garud’s *H* statistics (Garud et al. 2015) along the 2L chromosomal arm (*moving_garud_h* utility in *scikit-allel*; block length = 500 phased variants with 20% step). We performed the same calculations for the haplotypic diversity (*moving_haplotype_diversity* in *scikit-allel*). We calculated the Garud *H* and haplotypic diversity estimates in the *Rdl* locus, using a jack-knife procedure (Tukey 1958) (iterative removal of individual haplotypes without replacement) to calculate the mean and SE of each statistic.

### Karyotyping of 2La Inversions

In order to assign karyotypes of the 2La inversion in all specimens from *Ag1000G* Phase 2, we used known 2La karyotypes from Phase 1 as a reference (Miles et al. 2017), and analyzed genotype frequencies within the inversion by principal component analysis (PCA). Specifically, we retrieved the genotype frequencies of 1,142 specimens from *Ag1000G* Phase 2, 765 of which were also present in Phase 1 and had been previously karyotyped for this inversion (Miles et al. 2017); and selected 10,000 random SNPs (biallelic, shared between more than two samples, phased, segregating in at least one population, and located within the 2La inversion 2L:20524058–42165532). SNPs fitting these criteria were selected using the *scikit-allel* Python library, and the PCA was performed using the *randomized_pca* utility (with Patterson scaling).

Manual inspection of the principal components (supplementary material SM9, Supplementary Material online) showed that PC1 (6.35% of variance explained) was sufficient to discriminate between known karyotypes from Phase 1 using a clear-cut threshold (2La/2La, 2La/2L+^a^, and 2L+^a^/2L+^a^). We determined the optimal classification thresholds using the C-Support Vector classification method (SVC, a method for supervised learning) implemented in the *scikit-learn* 0.21.3 Python library (Pedregosa et al. 2011). Specifically, we used the *SVC* function in *scikit-learn* (*svm* submodule) to train a classifier with known karyotypes from Phase 1 (765 observations) and the main principal components of the PCA analysis (10 variables), using a linear kernel and *C* = 1. The selected thresholds were able to classify Phase 1 data into each of the three categories (2La/2La, 2La/2L+^a^, and 2L+^a^/2L+^a^) with 100% accuracy (as per the classifier *score* value), precision, and recall (calculated using the *classification_report* function from the *scikit-learn metrics* submodule).

### Phylogenetic Analysis of Haplotypes

We obtained genomic alignments of SNPs located from four regions around the *Rdl* locus, at the following coordinates: 1) 5′ start of the gene (2L:25363652±10,000 kb, 696 variants), 2) 3′ end of the gene (2L:25434556±10,000 kb, 428 variants), 3) unadmixed region 1 Mb upstream of *Rdl* (2L:24363652 + 20,000 kb; 2,903 variants; inside of the 2La inversion), and 4) unadmixed region 1 Mb downstream of *Rdl* (2L:26434556 + 20,000 kb, 2,594 variants; inside of the 2La inversion). These alignments were built from phased, biallelic variants within the aforementioned regions, obtained from *A. coluzzii* and *A. gambiae* (*Ag1000G* Phase 2), *A. arabiensis*, *A. quadriannulatus*, *A. melas*, and *A. merus*. We restricted our analysis to haplotypes pertaining to individuals homozygous for the 2La inversion (2La/2La and 2L+^a^/2L+^a^), totaling 1,684 haplotypes (out of 2,356 haplotypes in the original data set, obtained from 1,178 specimens). Invariant sites were removed from the alignments using *snp-sites* 2.3.3 (Page et al. 2016). All alignments are available in supplementary material SM10, Supplementary Material online.

Each genomic alignment was then used to compute maximum-likelihood phylogenetic trees using *IQ-TREE* 1.6.10 (Nguyen et al. 2015)*.* The best-fitting nucleotide substitution model for each alignment was selected using the *TEST* option of *IQ-TREE* and according to the Bayesian Information Criterion (BIC), which suggested the GTR substitution matrix with ascertainment bias correction, four gamma (*Γ*) rate categories, and empirical state frequencies observed from the alignment (F) (i.e., the *GTR+F+ASC+G4* model in *IQ-TREE*). We calculated branch statistical supports using the UF bootstrap procedure (Minh et al. 2013; Hoang et al. 2018) and refined the tree for up to 10,000 iterations, until convergence was achieved (correlation coefficient ≥ 0.99).

Tree visualizations were created in R, using the *plot.phylo* function from the *ape* 5.3 library (Paradis and Schliep 2019) and *stringr* 1.4.0 (Wickham 2019). Each phylogeny was midpoint-rooted with *phytools* 0.6–60 (Revell 2012) (*midpoint.root*), and branch lengths in figure 6 were constrained for display purposes (5 × 10^−5^ to 5 × 10^−3^ per-base substitutions range; unmodified trees available in supplementary material SM11, Supplementary Material online).

### Interspecific Introgression with Patterson’s *D* Statistic

We analyzed the signals of introgression along the 2L chromosomal arm using Patterson’s *D* statistic (Durand et al. 2011; Patterson et al. 2012). This statistic requires allele frequencies in four populations (A, B, C, and O) following a predefined (((A, B),C),O) phylogeny, where A, B, and C are populations with possible introgression events, and O is an unadmixed outgroup. Then, *D *>* *0 if there is an excess of allele frequency similarities between A and C (which means either A → C or C → A introgression) and *D *<* *0 for excess of similarity between B and C (B → C or C → B introgression) (Durand et al. 2011; Patterson et al. 2012). We calculated Patterson’s *D* along blocks of adjacent variants in the 2L chromosomal arm (block length = 10,000 variants, with 20% step length; phased variants only) using the *moving_patterson_d* utility in *scikit-allel*. We also calculated *D* in the *Rdl* locus (2L:25363652–25434556), and estimated its deviation from the null expectation (no introgression: *D *=* *0) with a block-jackknife procedure (block length = 100 variants; *average_patterson_d* in *scikit*-*allel*). We then used these jack-knifed estimates to calculate the SE, *Z*-score, and the corresponding *P* value from the two-sided *Z*-score distribution.

Using the procedure described above, we performed multiple analyses of introgression between combinations of populations fitting the (((A, B),C),O) phylogeny. For each analysis, we selected A, B, C, and O populations according to two criteria: 1) which interspecific introgression event was under test (*A. gambiae ∼ A. coluzzii* or *A. coluzzii ∼ A. arabiensis*); 2) homozygous karyotypes of the 2La inversion within which *Rdl* is located (given that it introduces a strong effect on genotype frequencies across the entire *A. gambiae* species complex; Fontaine et al. 2015) and the resistance haplotype in question; and 3) exclude populations with high frequencies of hybrids, with controversial species identification, or with extreme demographic histories (Guinea-Bissau, The Gambia, and Kenya) (Miles et al. 2017; Vicente et al. 2017). Following these criteria, we then tested the presence and direction introgression between the combinations of populations specified below.

First, we tested the *A. coluzzii* ∼ *A. arabiensis* introgression of the *296S* haplotype in inverted genomes (2La/2La homozygotes; fig. 7*A* and supplementary material SM12, Supplementary Material online). We performed two versions of this test, using either *A. coluzzii* or *A. arabiensis* as donors (population C), which can give an indication of the population of origin of the *296S* mutation. First, we tested the *A. arabiensis* → *A. coluzzii* hypothesis using: 1) *296S* homozygous *A. coluzzii* from Burkina Faso as population A; 2) *wt* homozygous *A. coluzzii* from Burkina Faso as population B; 3) *A. arabiensis* and *A. merus* specimens as multiple C populations (donors) C, treating *296S* and *wt* homozygous specimens as different populations; and 4) *A. epiroticus* and *A. christyi* as population O. Second, we tested the *A. coluzzii* → *A. arabiensis* hypothesis but switching the position of *A. arabiensis* (now population A and B, for *296S* and *wt*, respectively) and *A. coluzzii* populations (now population C, together with the *A. merus* negative control). Under this setup, we expect to see evidence of introgression between *296S A. coluzzii* and *296S A. arabiensis* in both tests (positive controls), but a positive result with any of the *wt* comparisons can indicate that *296S* haplotypes in either species is more similar to *wt* from the other (and hence, the second species is the species of origin). A detailed account of all comparisons, populations, and complete statistical reports are available in supplementary material SM12, Supplementary Material online.

We performed the same series of tests for the *A. gambiae* ∼ *A. coluzzii* introgression of the *296G* cluster in individuals without the 2La inversion (2L+^a^/2L+^a^ homozygotes; fig. 7*B* and supplementary material SM13*A* and *B*, Supplementary Material online) and with the 2La inversion (supplementary material SM13*C* and *D*, Supplementary Material online). In these tests, homozygous individuals from various *A. gambiae* and *A. coluzzii* populations were alternatively used as groups A/B (A if *296G*, B if *wt*) and C (*296G* and *wt*, separately); and *wt* outgroups were selected according to their 2La karyotype (2L+^a^/2L+^a^: *A. quadriannulatus* and *A. melas*; 2La/2La: *A. merus*). A detailed account of all comparisons, populations, and complete statistical reports are available in supplementary material SM13, Supplementary Material online.

### Sequence Divergence between 2La Karyotypes

To ascertain whether *296G* karyotypes from 2La chromosomes were introgressed from a 2L+^a^ background, we calculated the absolute sequence divergence (*Dxy*; Takahata and Nei 1985) around the *Rdl* locus between all combinations of the following groups of haplotypes: 1) between *296G*-carrying haplotypes from 2L+^a^/2L+^a^ homozygotic genomes, 2) *wt* haplotypes from 2La/2La, 3) *296G* haplotypes from 2La/2La, and 4) *wt* haplotypes from 2La/2La (fig. 8). *Dxy* estimates were calculated along the 2L arm using the *windowed_divergence* utility in *scikit-allel* (window size = 20,000 bp with 10% overlap). At each window, we also calculated the ratio between the following *Dxy* estimates: 1) *296G*-2L+^a^ ∼ *wt*-2La/*296G*-2L+^a^ ∼ *wt*-2L+^a^; and 2) *296G*-2La ∼ *wt*-2La/*296G*-2La ∼ *wt*-2L+^a^. Thus, windows with ratios >1 are more similar to the *wt*-2L+^a^ background, and windows with ratios <1 are more similar to the *wt*-2La background.

### Alignment of *Rdl* Orthologs

We retrieved *Rdl* orthologs from the following species of the Culicidae family (available in Vectorbase): *A. gambiae*, *A. arabiensis*, *A. melas*, *A. merus*, *A. christyi*, *A. epiroticus*, *A. minimus*, *A. culicifacies*, *A. funestus*, *A. stephensi*, *A. maculatus*, *A. farauti*, *A. dirus*, *A. atroparvus*, *A. sinensis*, *A. albimanus*, *A. darlingi*, *Aedes aegypti*, *Aedes albopictus*, and *Culex quinquefasciatus*. We retained 1) those orthologs that resulted in complete predicted peptides (defined as having the same start and end codons as the *A. gambiae Rdl*), and 2) the longest isoform per gene (except for *A. gambiae*, where all three isoforms were retained). These sequences were aligned using *MAFFT* 7.310 (1,000 rounds of iterative refinement, G-INS-i algorithm) (Katoh and Standley 2013). Pairwise sequence identity between peptide sequences was calculated using the *dist.alignment* function (with an identity distance matrix, which was then converted to a pairwise identities) from the *seqinr* 3.4–5 library (Charif and Lobry 2007), in R 3.6.1 (R Core Team 2017). Pairwise *d*_N_/*d*_S_ ratios were calculated from a codon-aware alignment of CDS sequences, using the *dnds* function from the *ape* 5.3 R library (Paradis et al. 2004). The codon-aware alignment of full-length CDS was obtained with PAL2NAL (Suyama et al. 2006), using the peptide alignment as a reference. Tables of pairwise identity and *d*_N_/*d*_S_ values have been created with *pheatmap* 1.0.12 (Kolde 2019).

### Homology Modeling and Automated Ligand Docking

The structure of human GABA_A_ receptor bound with picrotoxin (PDB accession: 6HUG) provided the template for generating a homology model of the homopentameric *A. gambiae* RDL receptor (UniProtKB accession: Q7PII2). Sequences were aligned using *Clustal Omega* (Sievers et al. 2011), and 50 homology models were generated using *MODELLER* 9.23 (Eswar et al. 2006). A single best model was chosen based on the internal scoring values from *MODELLER* and by visually inspecting models in *Swiss-PdbViewer* (Guex et al. 1999) to eliminate candidates with structural problems. The *A296G* and *A296S* mutants were generated using *Swiss-PdbViewer* to introduce the amino acid substitutions and to energy minimize the resulting structures using 50 steps of conjugate gradient energy minimization. The pore radii of the channel models were calculated using *HOLE* 2.0 (Smart et al. 1996). The 3D structure of dieldrin was generated ab initio using *MarvinSketch* 19.22 of the ChemAxon suite (ChemAxon 2019). *AutoDockTools* 1.5.6 (Morris et al. 2009) was used to define rotatable bonds and merge nonpolar hydrogens. Automated ligand docking studies with the wild-type GABA receptor model were performed using *AutoDock Vina* 1.1.2 (Trott and Olson 2009) with a grid of 20 × 20 × 20 points (1Å spacing) centered on the channel pore. Figures were produced using *PyMOL* (Schrödinger 2015).

### Availability of Code and Data

Python (3.7.4) and R scripts (3.6.1) to reproduce all analyses in this article are available on GitHub: https://github.com/xgrau/rdl-Agam-evolution (last accessed June 6, 2020).

All genome variation data have been obtained from the publicly available repositories of the *Ag1000G* project Phase 2-AR1 (*Anopheles gambiae* 1000 Genomes Consortium 2017). Accession codes are available in supplementary material SM1, Supplementary Material online, and download instructions can be found in the above-mentioned GitHub repository.

## Supplementary Material

Supplementary data are available at *Molecular Biology and Evolution* online.

## Supplementary Material

msaa128_supplementary_data
