# Supplementary material for: Evolution of the Insecticide Target Rdl in African Anopheles Is Driven by Interspecific and Interkaryotypic Introgression
Source: Mol Biol Evol. 2020 May 21;37(10):2900–17. doi: 10.1093/molbev/msaa128 (PMC7530614; doi:10.1093/molbev/msaa128)

Supplementary Material 12

A) *A. arabiensis* as donor (C), 2La homozygotes

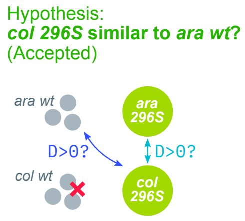

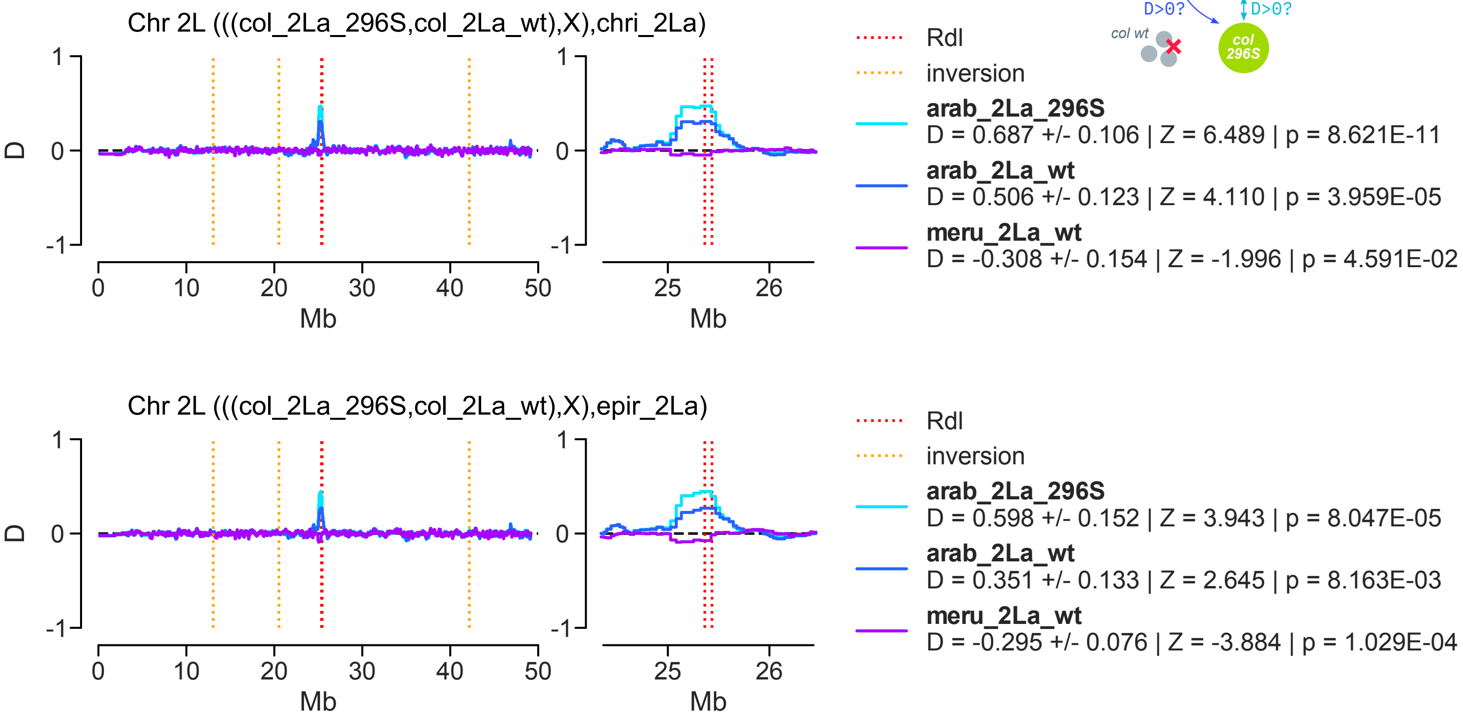

B) *A. coluzzii* as donor (C), 2La homozygotes

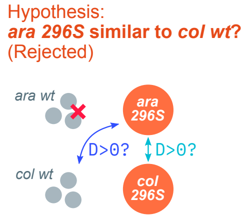

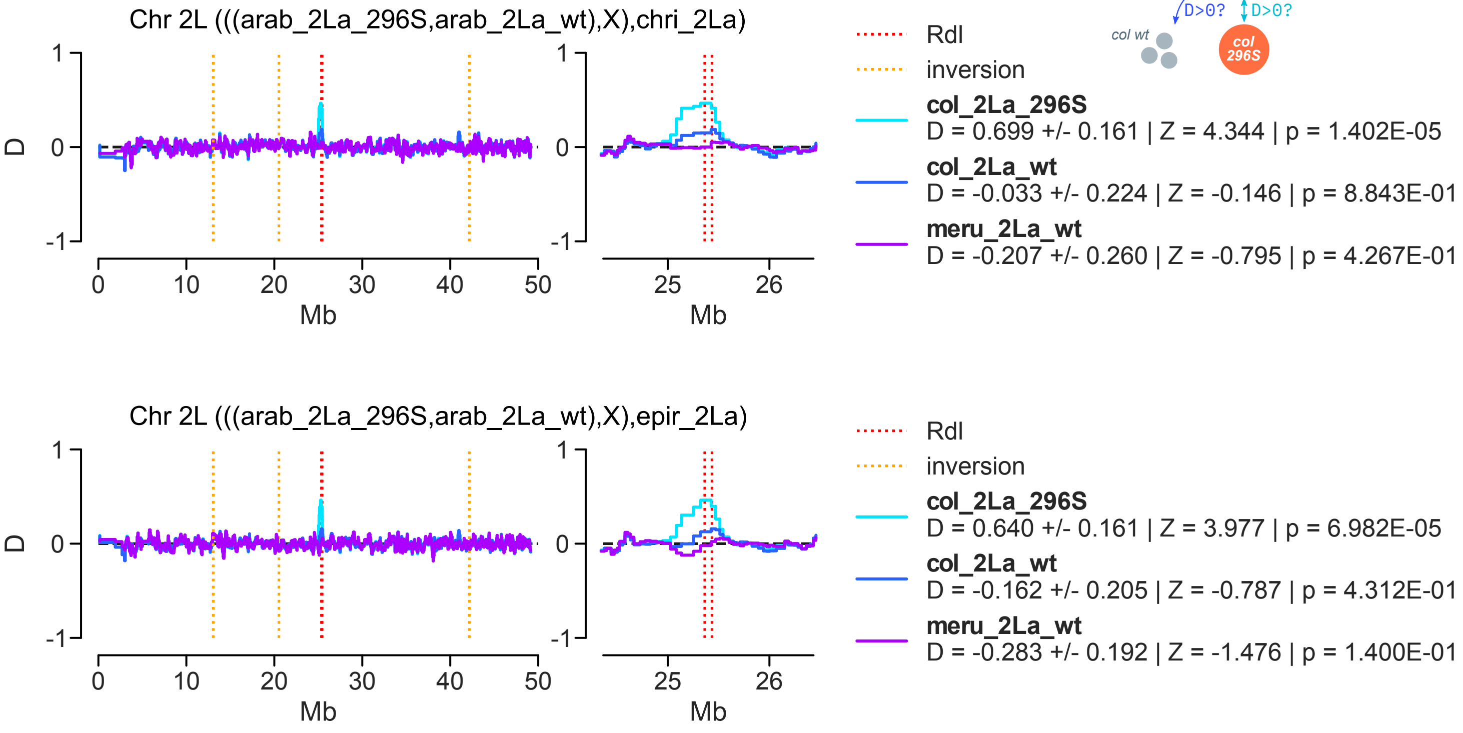

Supplement: msaa128_supplementary_data [file msaa128_supplementary_data.zip › sm12_admixtureD_aracol296S_2.pdf]
