# Supplementary material for: Evolution of the Insecticide Target Rdl in African Anopheles Is Driven by Interspecific and Interkaryotypic Introgression
Source: Mol Biol Evol. 2020 May 21;37(10):2900–17. doi: 10.1093/molbev/msaa128 (PMC7530614; doi:10.1093/molbev/msaa128)

Supplementary Material 13

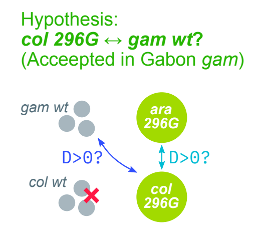

A) *A. gambiae* as donor (C), 2L+ª homozygotes

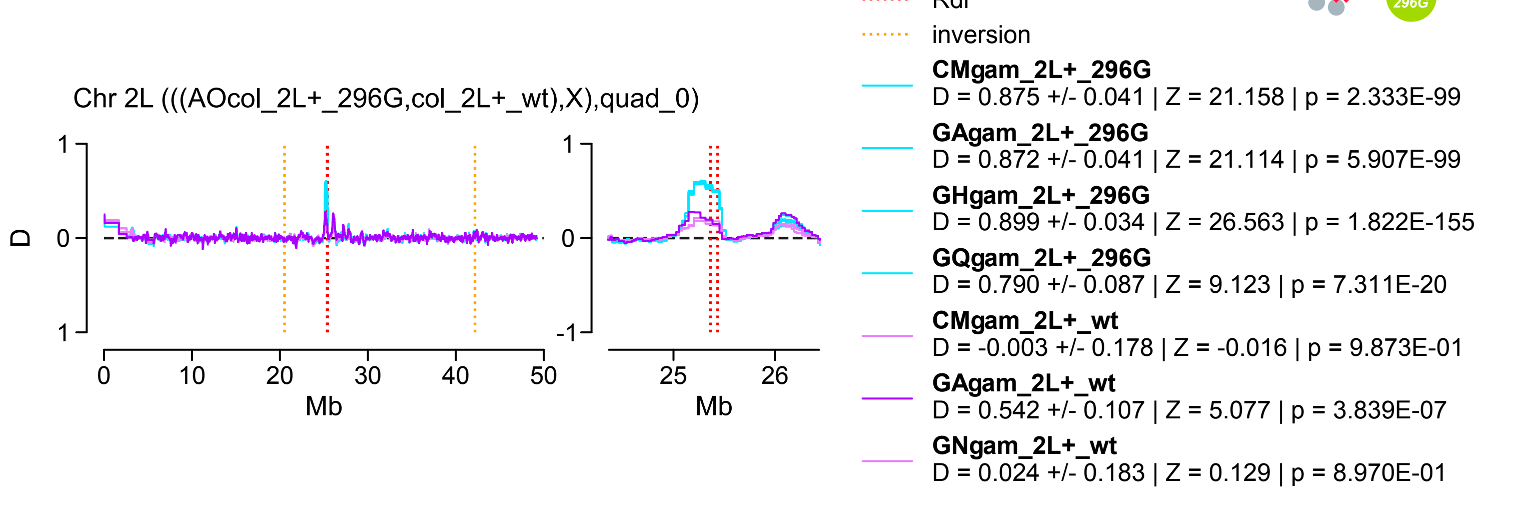

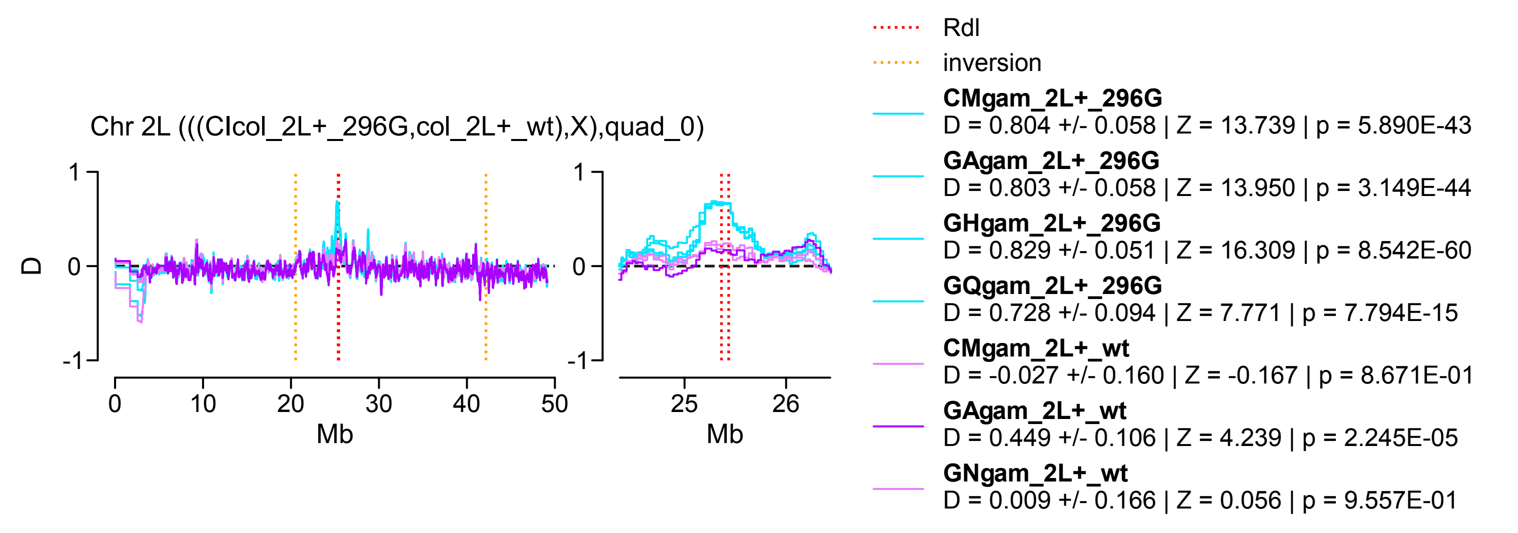

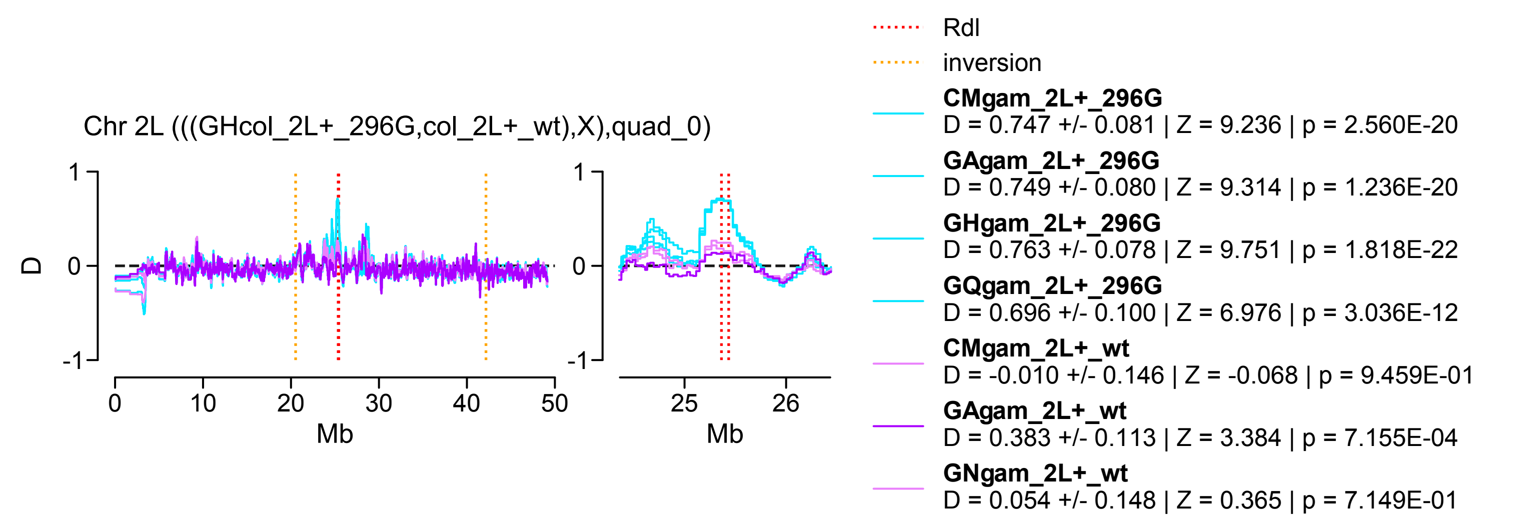

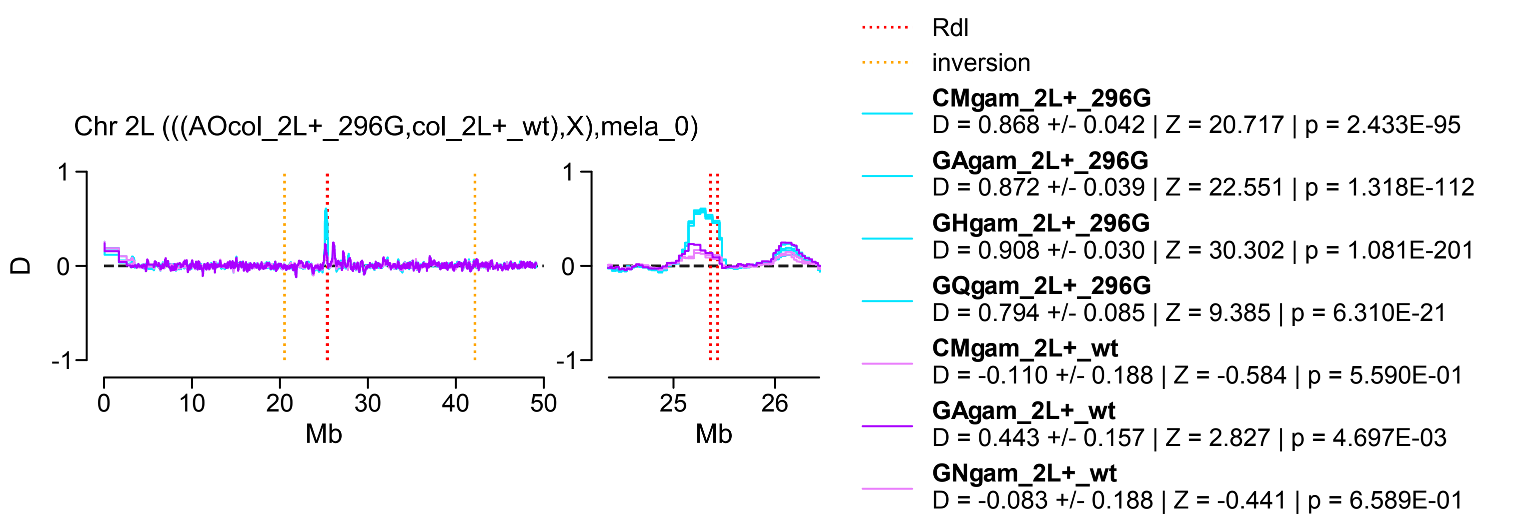

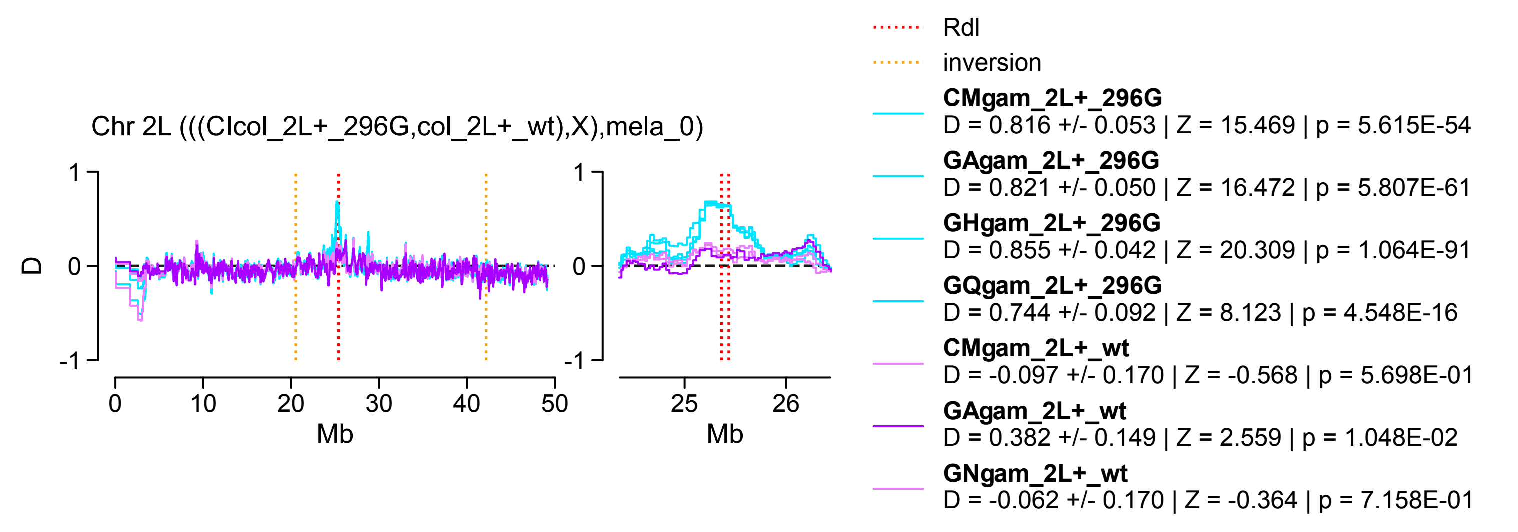

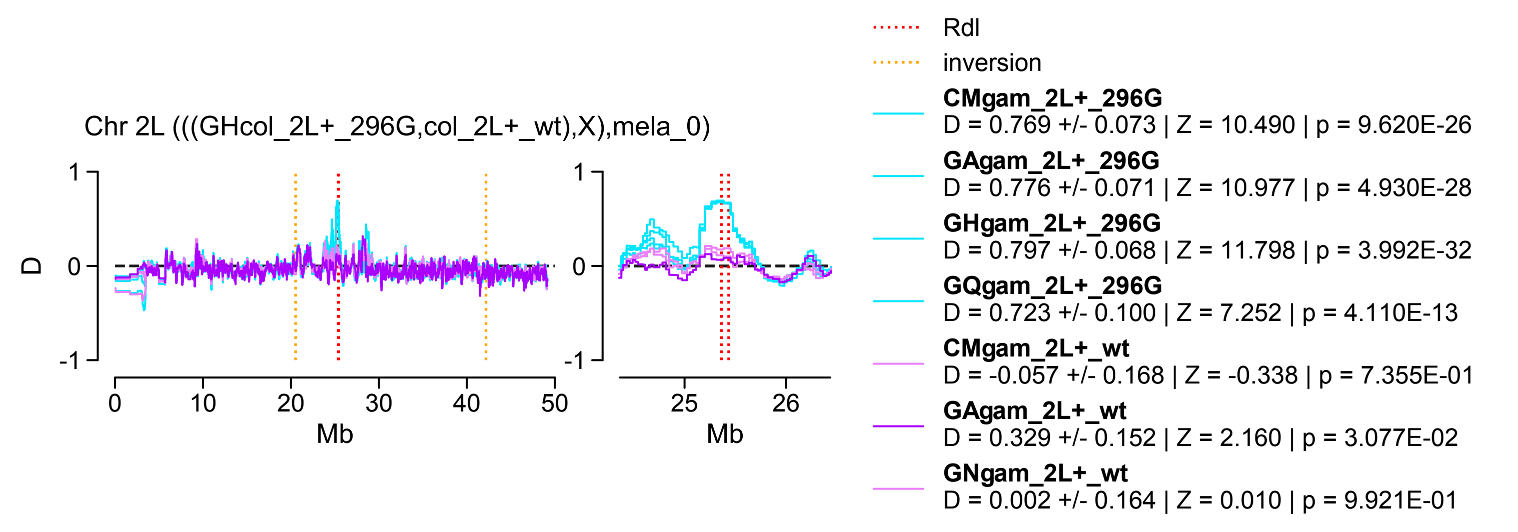

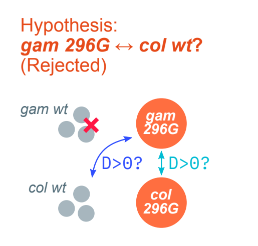

B) *A. coluzzii* as donor (C), 2L+ª homozygotes

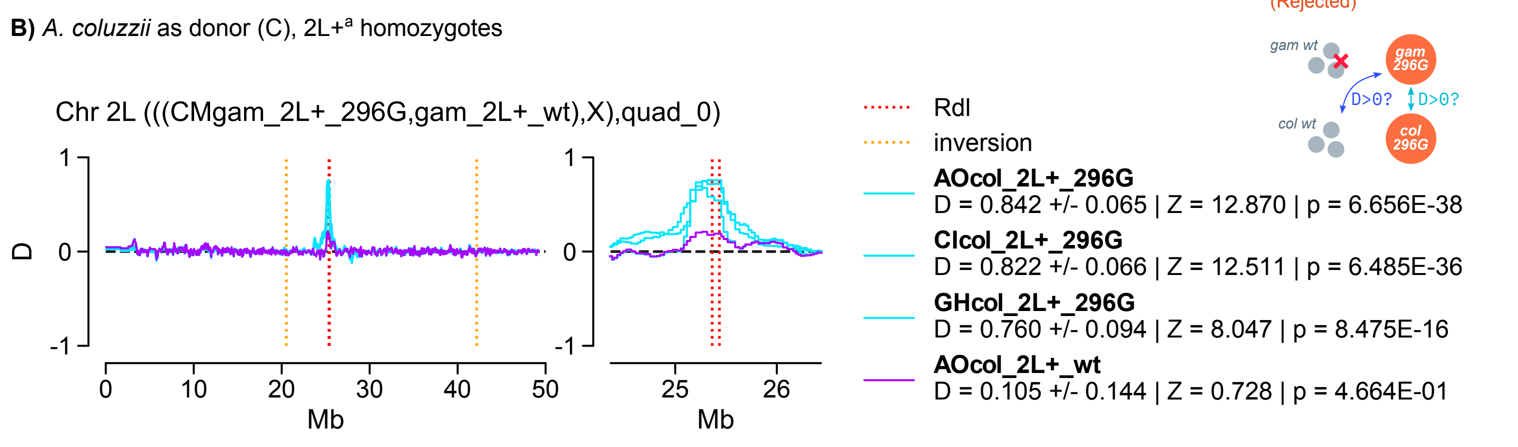

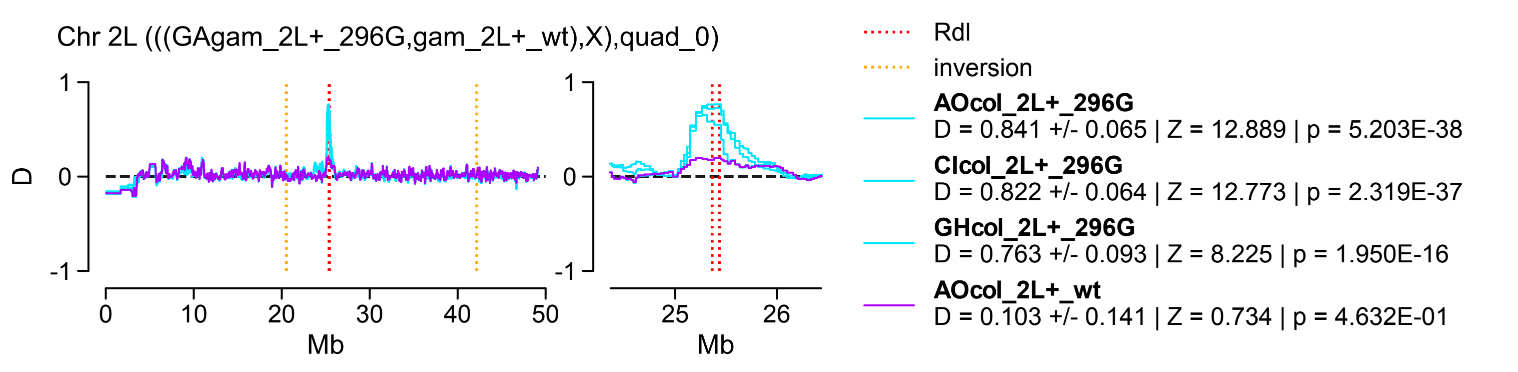

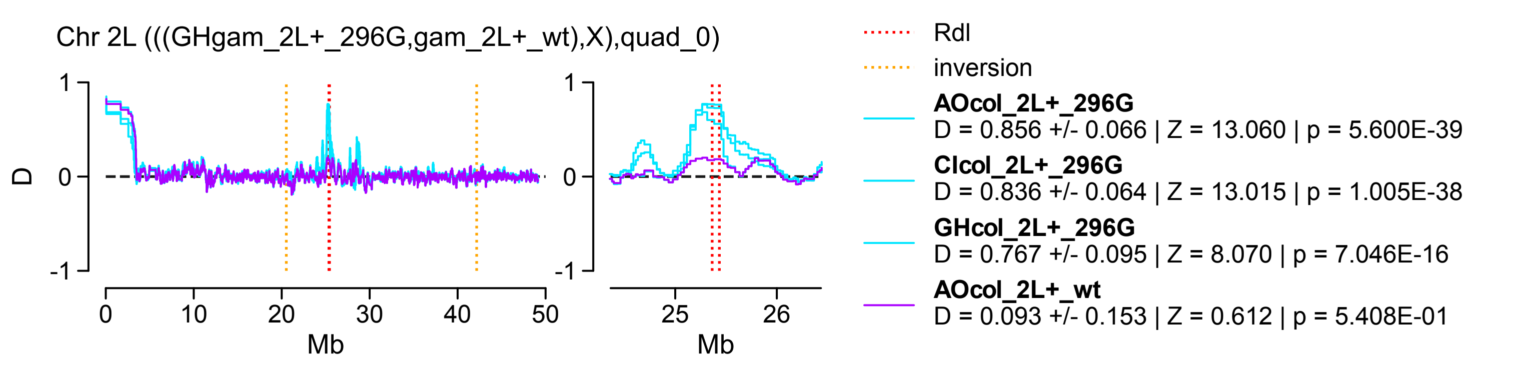

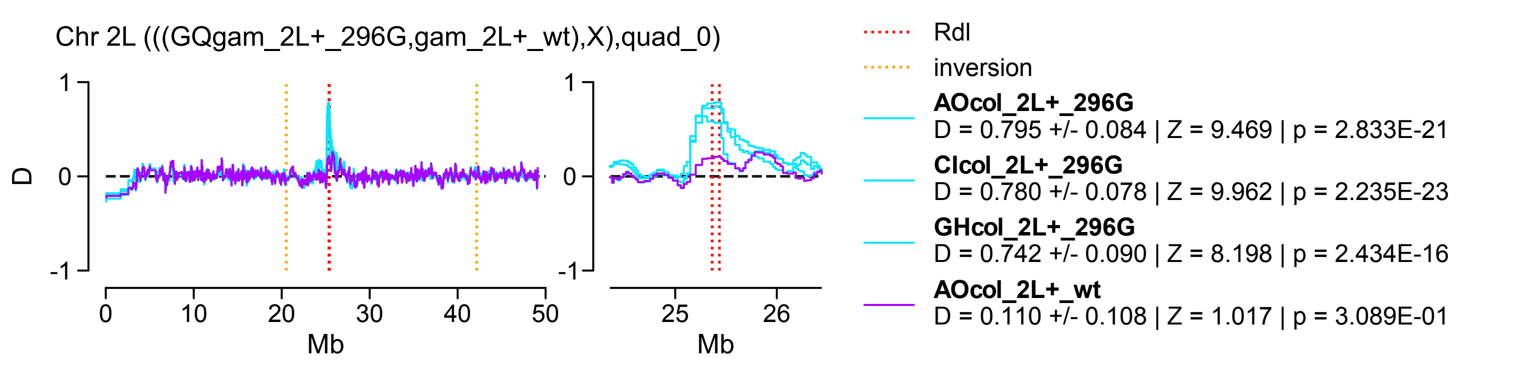

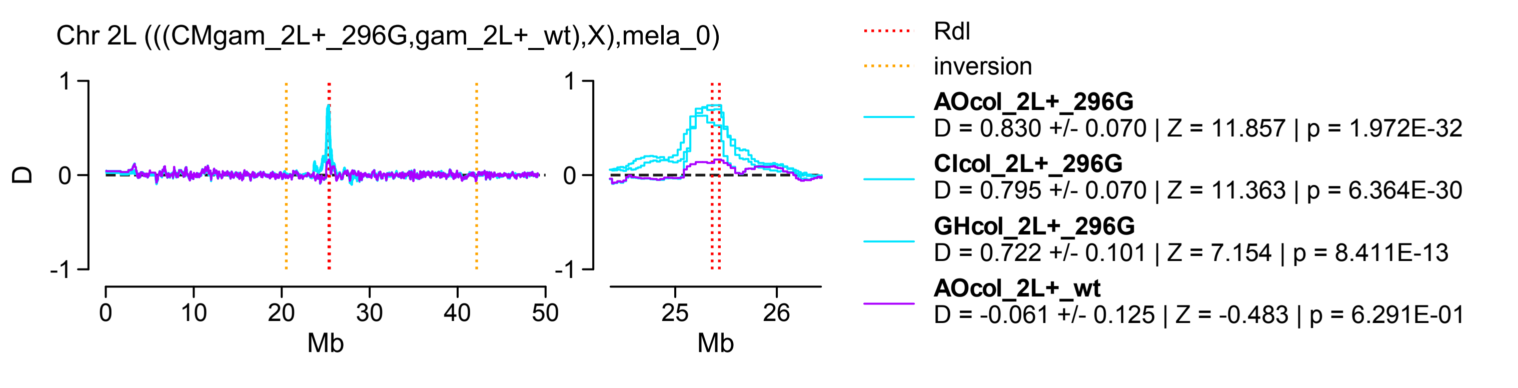

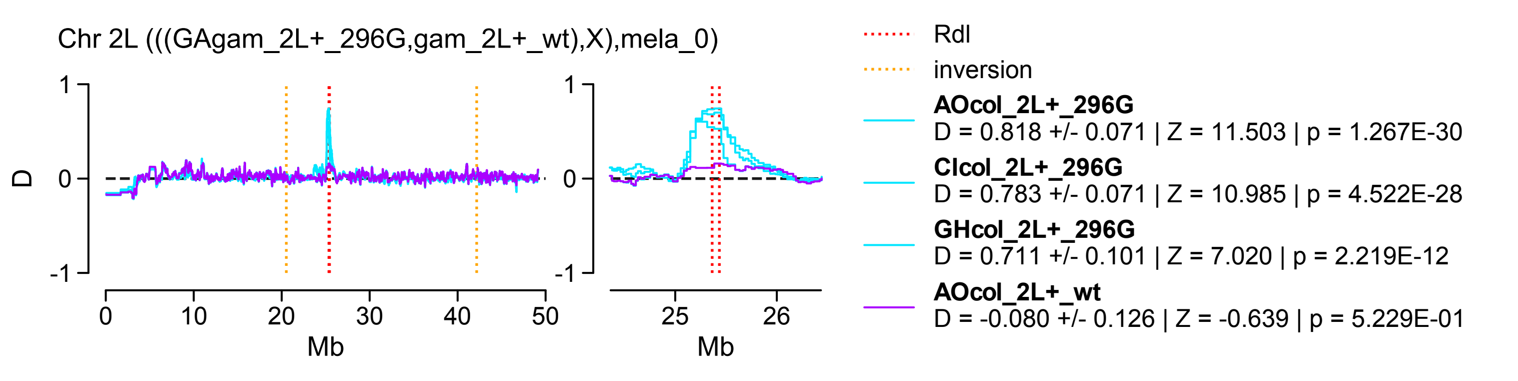

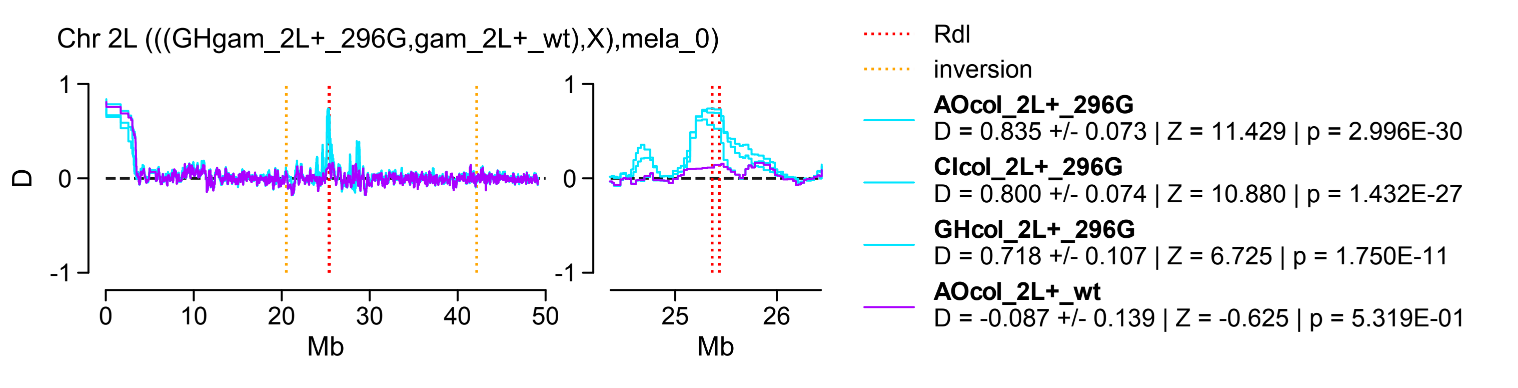

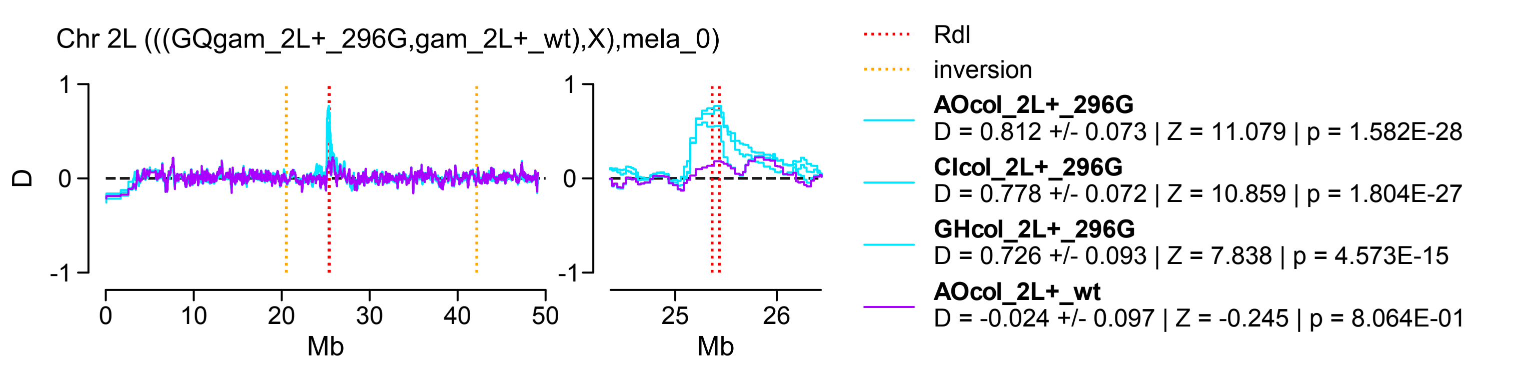

C) *A. gambiae* as donor (C), 2La homozygotes

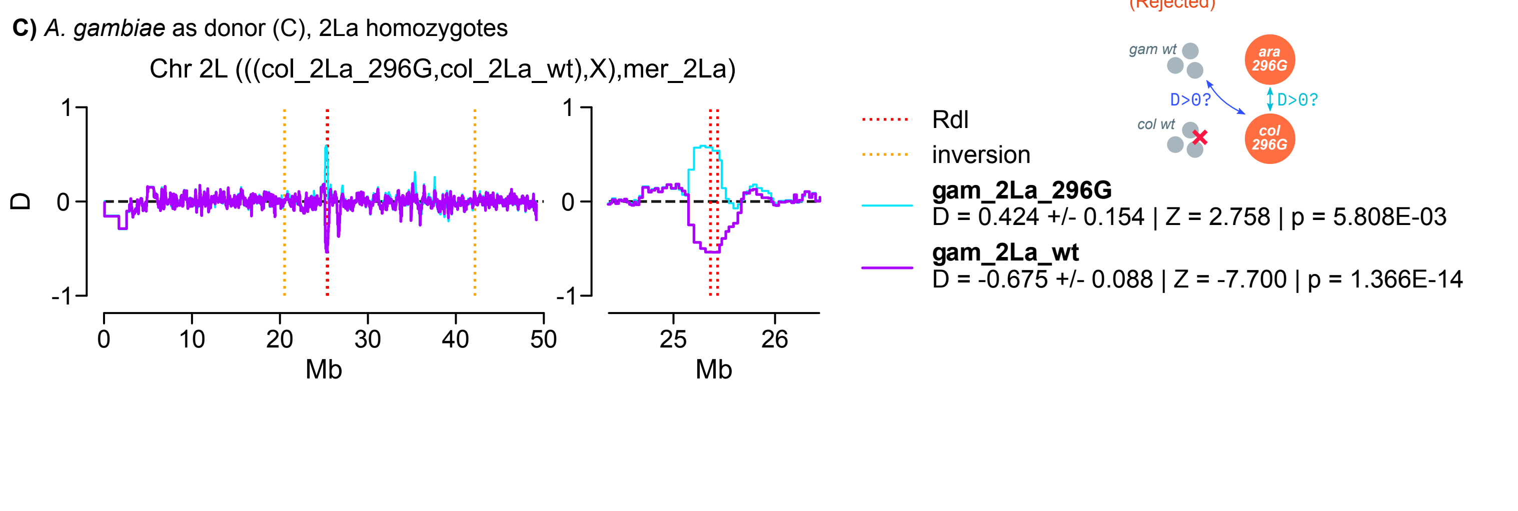

D) *A. coluzzii* as donor (C), 2La homozygotes

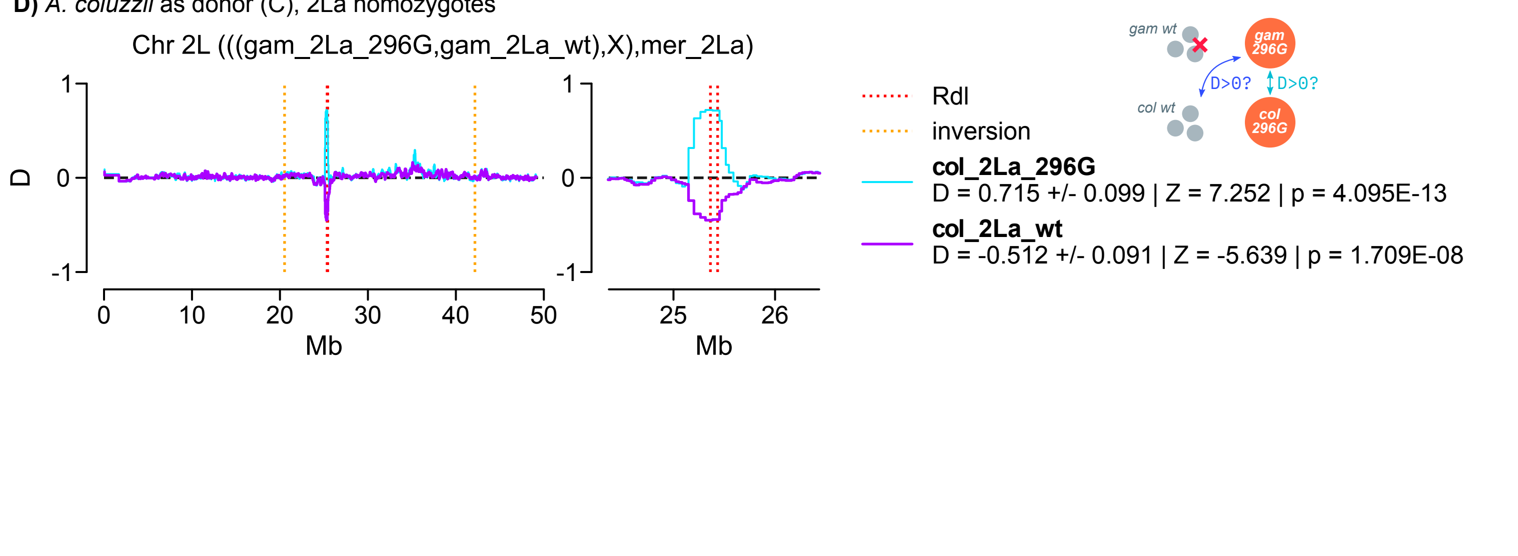

Supplement: msaa128_supplementary_data [file msaa128_supplementary_data.zip › sm13_admixtureD_gamcol296G_3.pdf]
