# Supplementary material for: Evolution of the Insecticide Target Rdl in African Anopheles Is Driven by Interspecific and Interkaryotypic Introgression
Source: Mol Biol Evol. 2020 May 21;37(10):2900–17. doi: 10.1093/molbev/msaa128 (PMC7530614; doi:10.1093/molbev/msaa128)

Supplementary Material 14

A) *EHH* decay 2L:25429236 +/- 150000, n=16623 vars

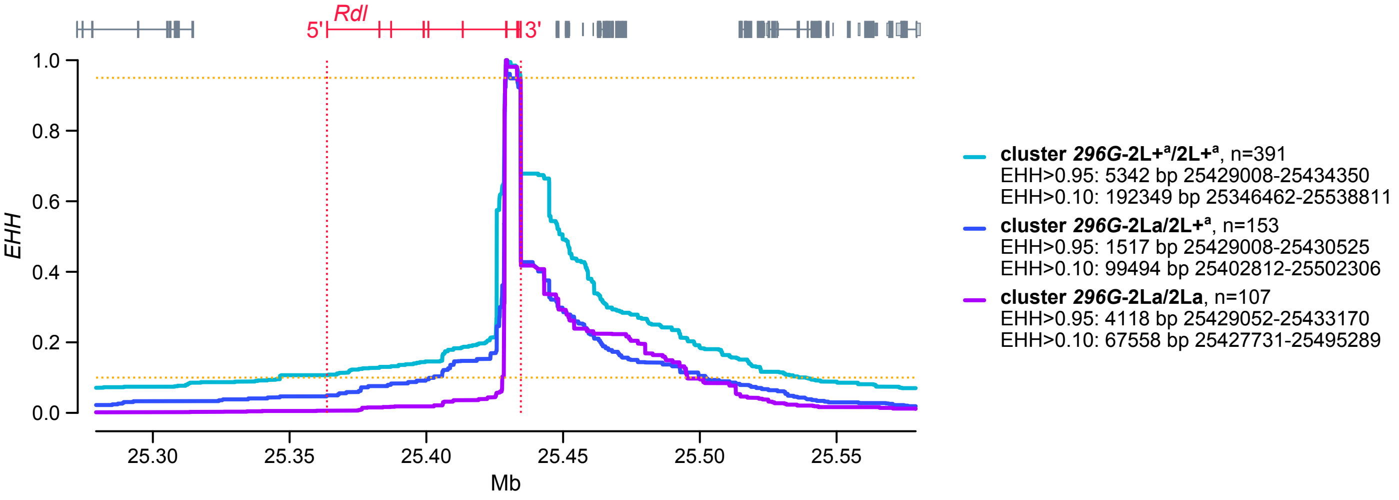

B) Haplotype diversity 2L:25429236 +/- 150000, n=16623 vars

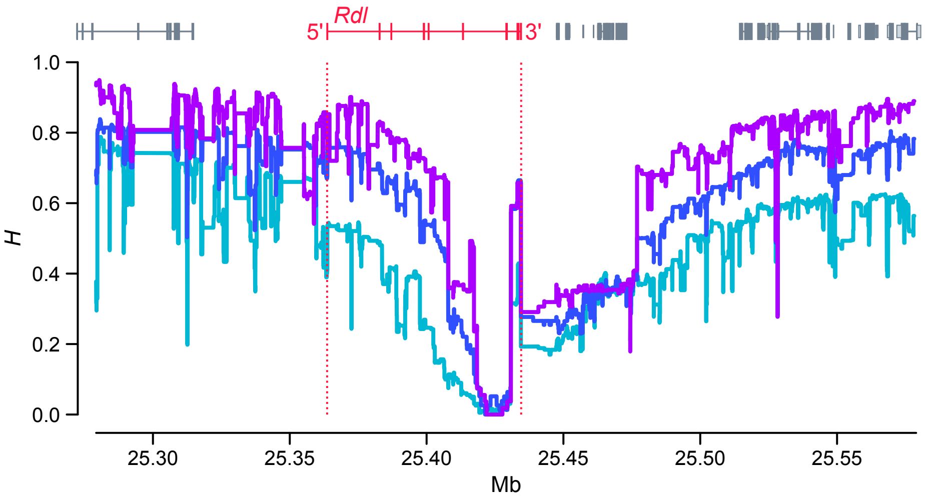

C) Sequence divergence between *296G* (2L+^{a} background) and *wt* (2L+^{a} or 2La) haplotypes

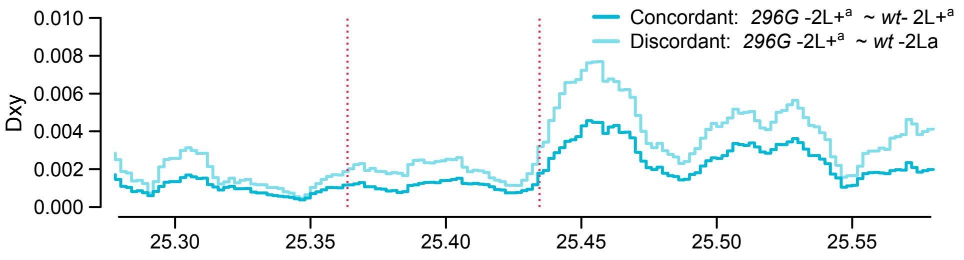

D) Sequence divergence between *296G* (2La background) and *wt* (2L+^{a} or 2La) haplotypes

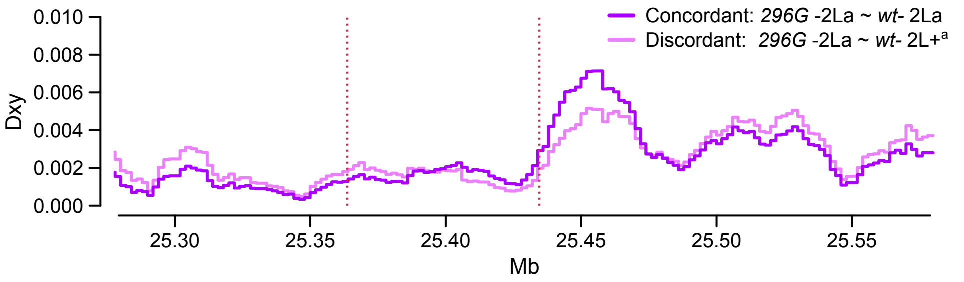

Supplement: msaa128_supplementary_data [file msaa128_supplementary_data.zip › sm14_hapdiv_haphom_2La4.pdf]
