# Supplementary material for: Evolution of the Insecticide Target Rdl in African Anopheles Is Driven by Interspecific and Interkaryotypic Introgression
Source: Mol Biol Evol. 2020 May 21;37(10):2900–17. doi: 10.1093/molbev/msaa128 (PMC7530614; doi:10.1093/molbev/msaa128)

Supplementary Material 15

A) *A. gambiae* 2L+^{a}/2L+^{a} ~ other

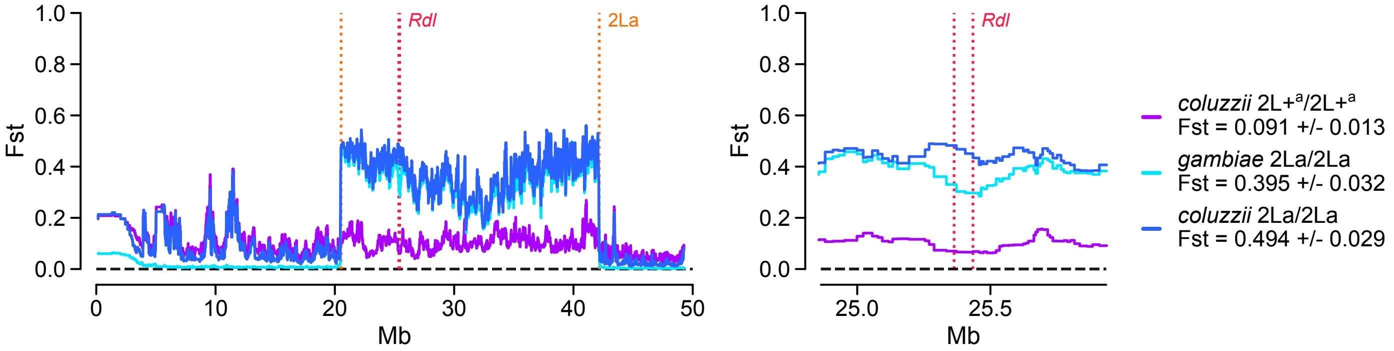

B) *A. gambiae* 2La/2La ~ other

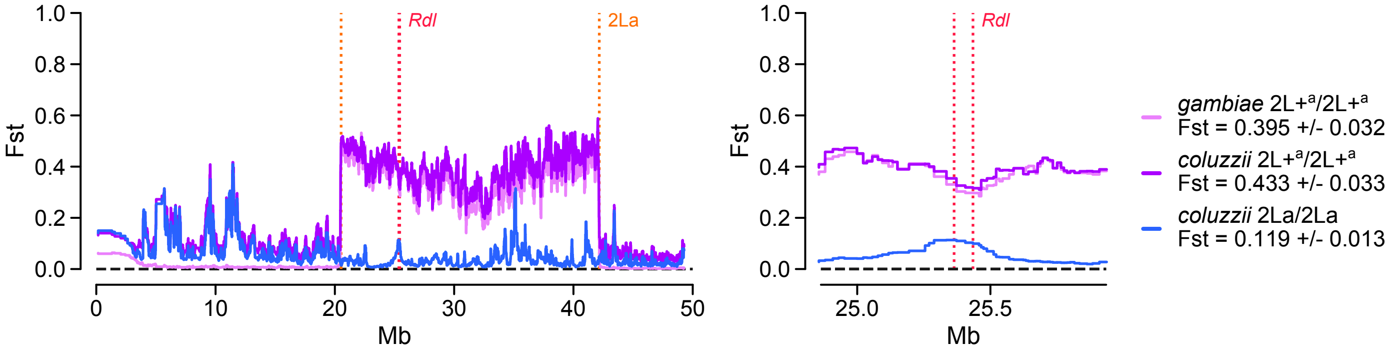

Supplement: msaa128_supplementary_data [file msaa128_supplementary_data.zip › sm15_differentiation_2La.pdf]
