# Supplementary material for: Evolution of the Insecticide Target Rdl in African Anopheles Is Driven by Interspecific and Interkaryotypic Introgression
Source: Mol Biol Evol. 2020 May 21;37(10):2900–17. doi: 10.1093/molbev/msaa128 (PMC7530614; doi:10.1093/molbev/msaa128)

Supplementary Material 6

A) Minimum spanning network of haplotypes in *Rdl*, colored by *Rdl* 296th codon genotype

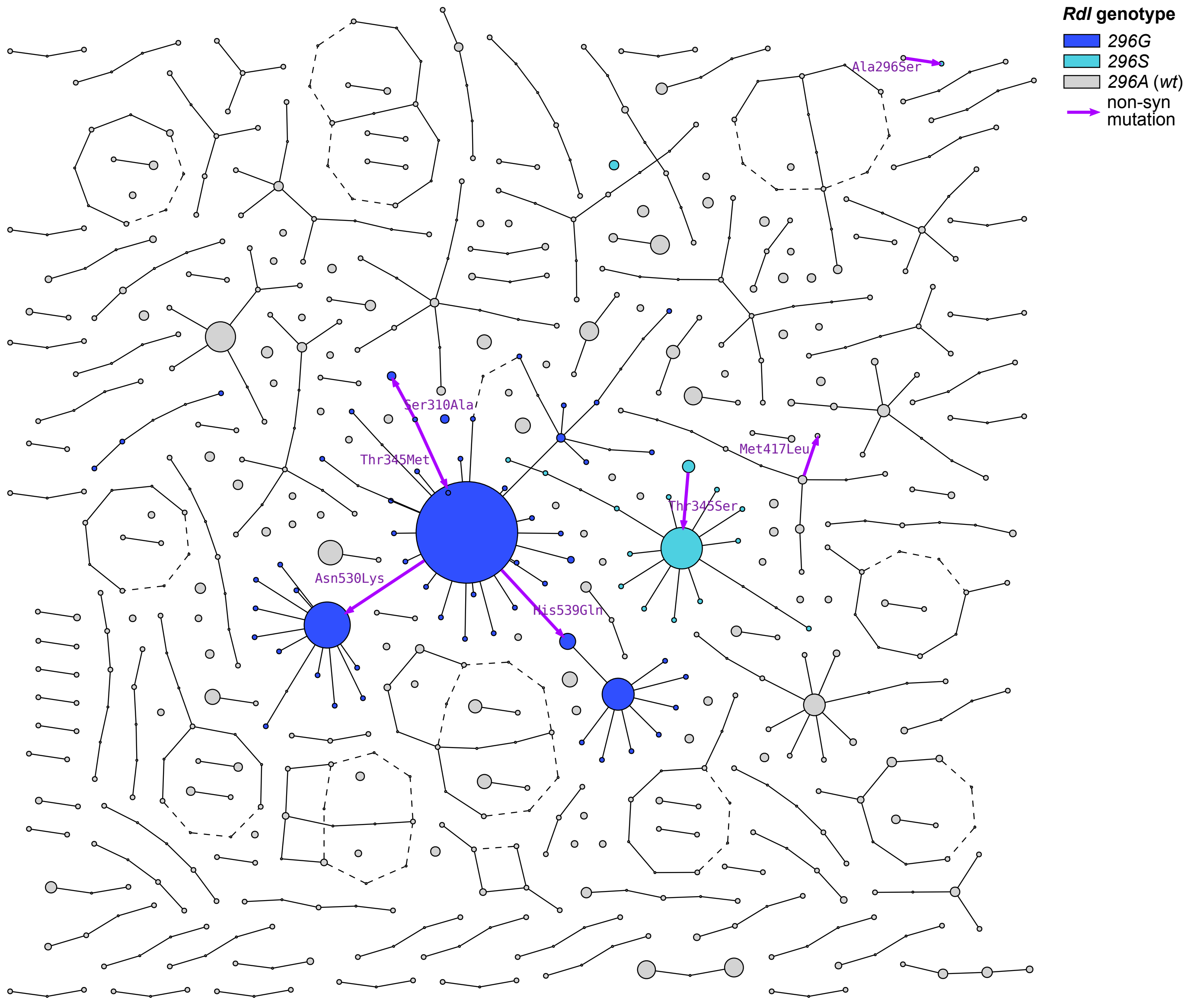

B) Minimum spanning network of haplotypes in *Rdl*, colored by *Vgsc* 995th codon genotype

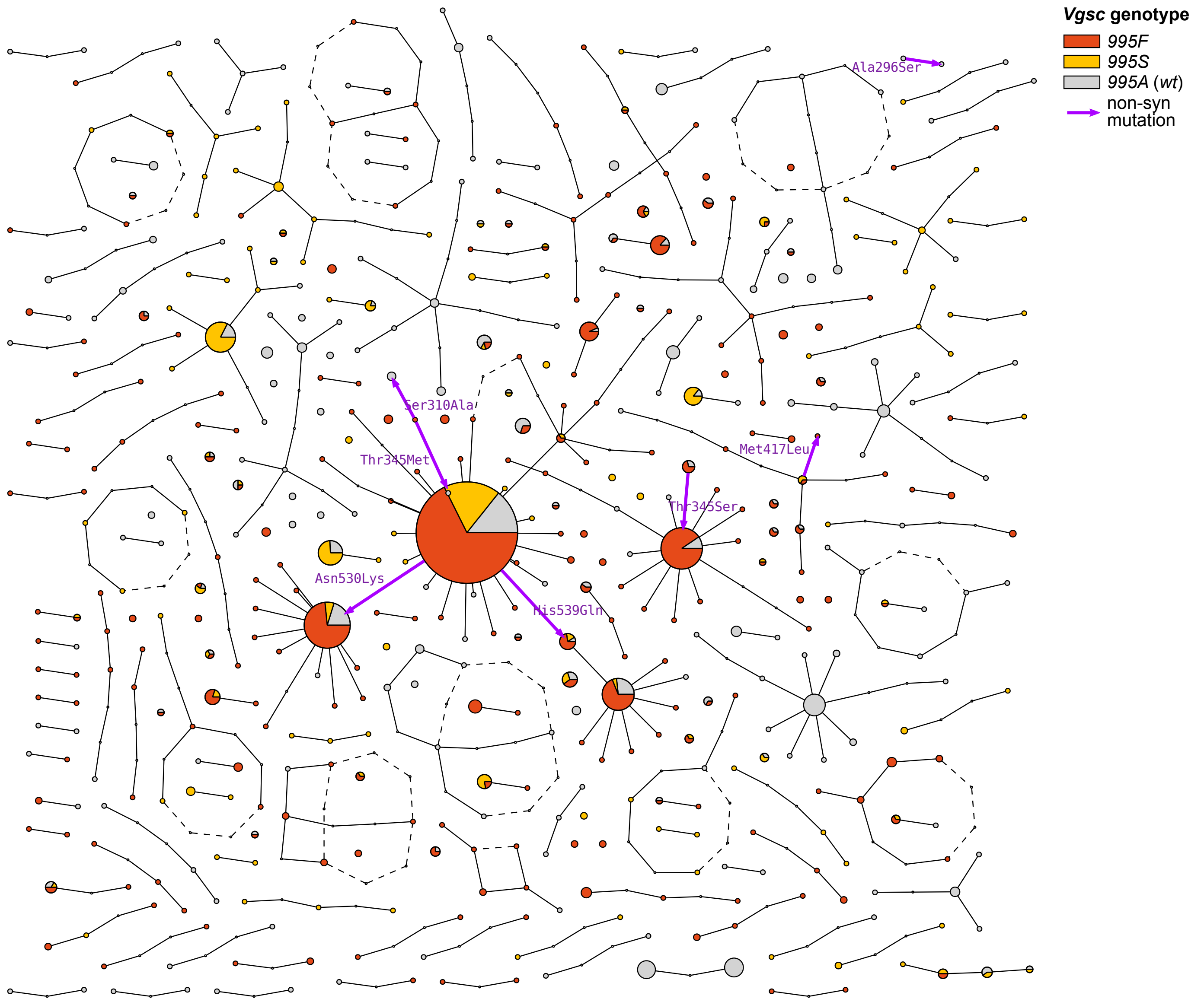

C) Minimum spanning network of haplotypes in *Rdl*, colored by species

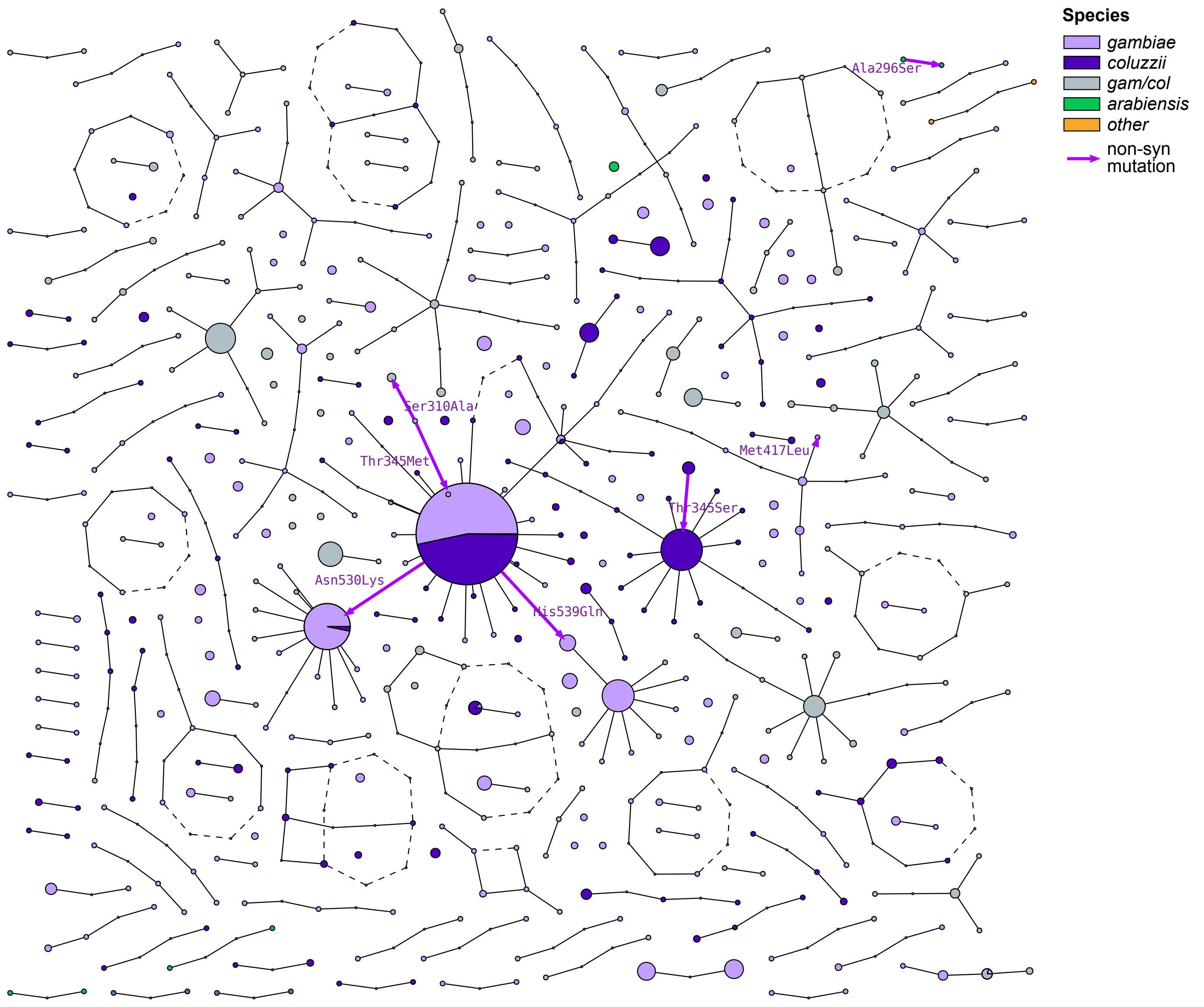

Supplement: msaa128_supplementary_data [file msaa128_supplementary_data.zip › sm6_haplotype_networks_Rdl.pdf]
