# Supplementary material for: Evolution of the Insecticide Target Rdl in African Anopheles Is Driven by Interspecific and Interkaryotypic Introgression
Source: Mol Biol Evol. 2020 May 21;37(10):2900–17. doi: 10.1093/molbev/msaa128 (PMC7530614; doi:10.1093/molbev/msaa128)

Supplementary Material 7

A) Linkage disequilibrium, Huff and Rogers *r*

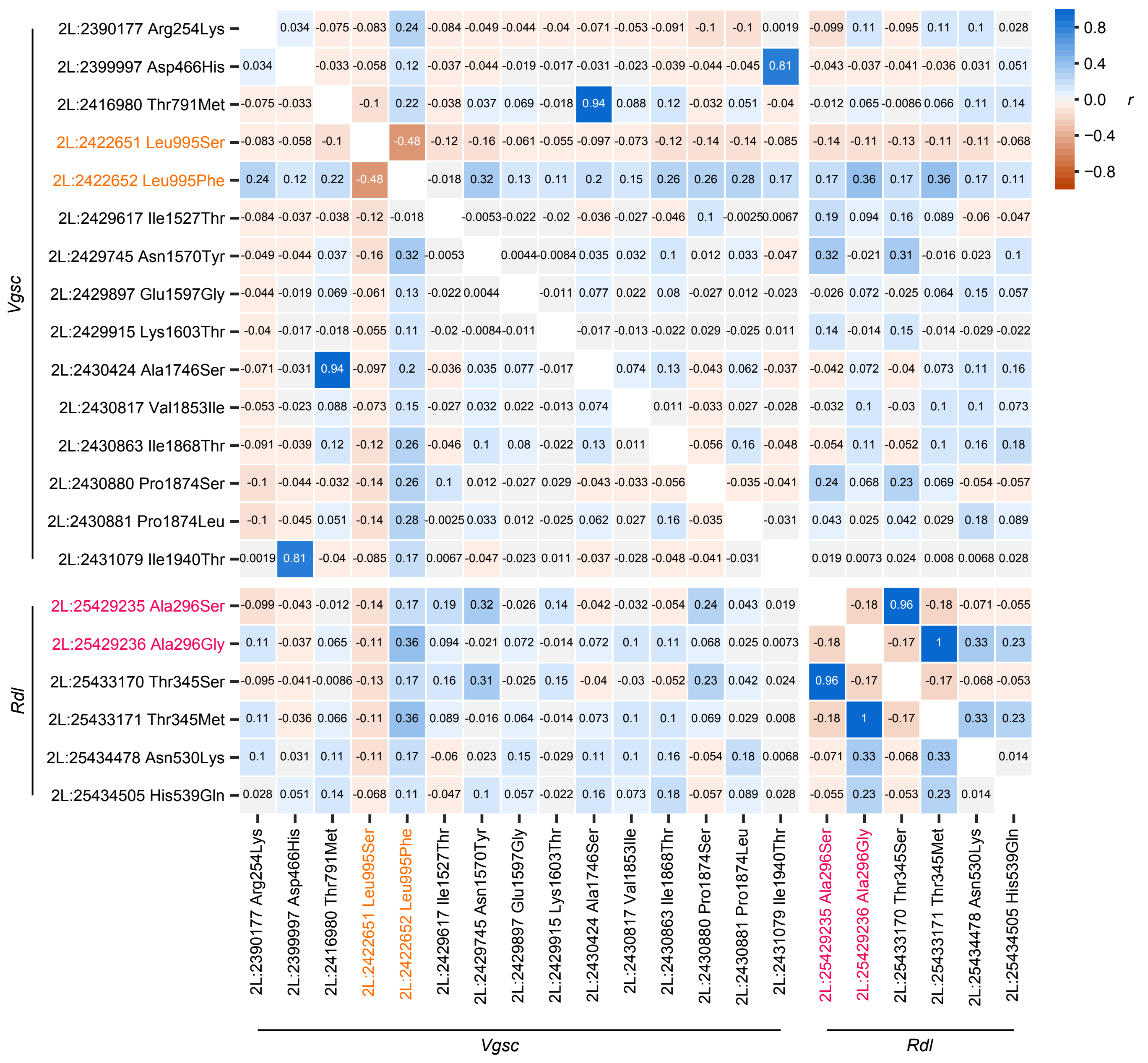

B) Linkage disequilibrium, Lewontin *D′*

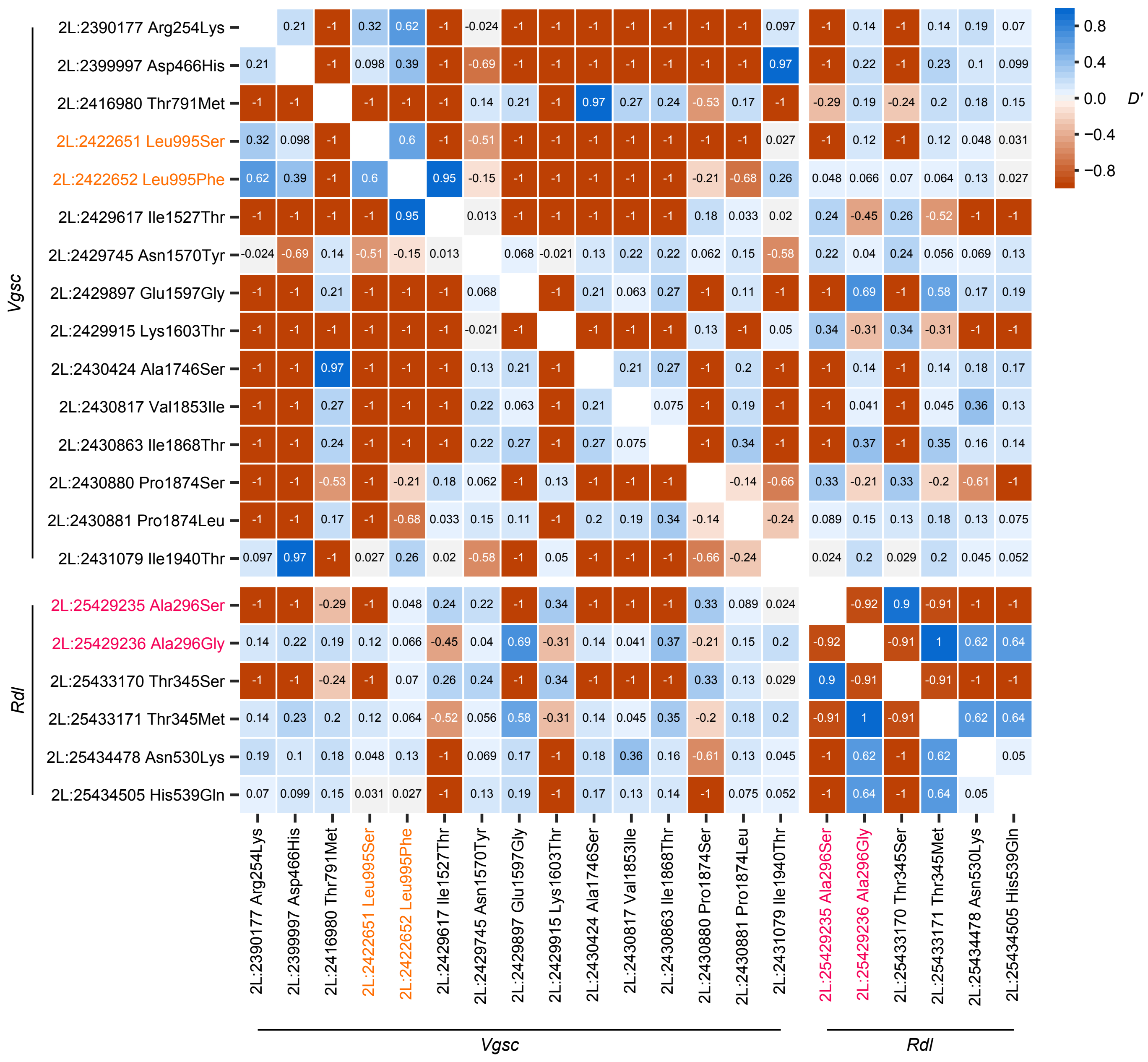

Supplement: msaa128_supplementary_data [file msaa128_supplementary_data.zip › sm7_LD_vgsc_rdl.pdf]
