# Supplementary material for: Evolution of the Insecticide Target Rdl in African Anopheles Is Driven by Interspecific and Interkaryotypic Introgression
Source: Mol Biol Evol. 2020 May 21;37(10):2900–17. doi: 10.1093/molbev/msaa128 (PMC7530614; doi:10.1093/molbev/msaa128)

Supplementary Material 8

A) Frequency *Vgsc* codon 995 mutations

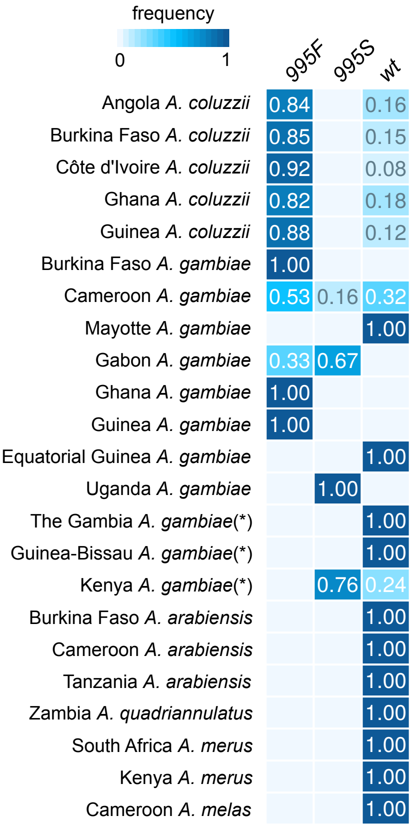

B) Frequency *Rdl* codon 296 mutations

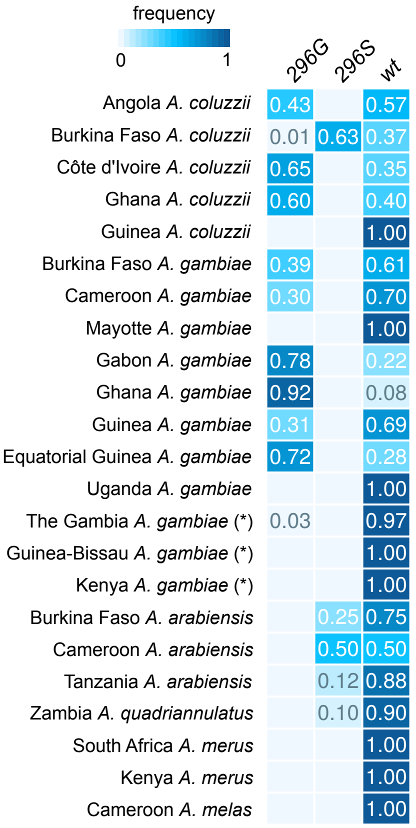

C) Geographical co-occurrence

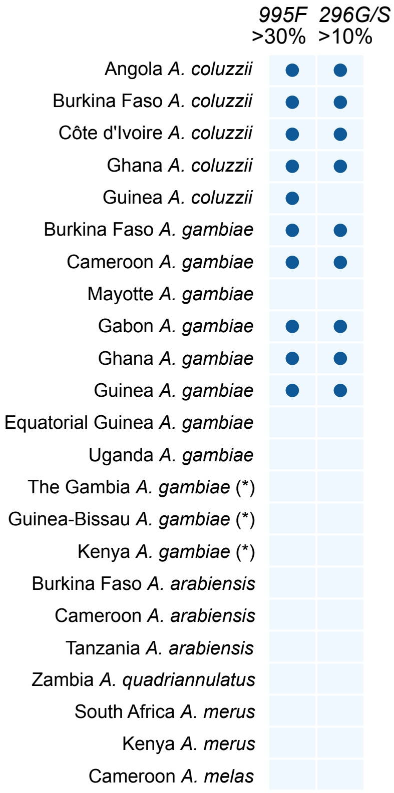

D) Overlap of *Rdl* codon 296 and *Vgsc* codon 995 alleles, per chromosome

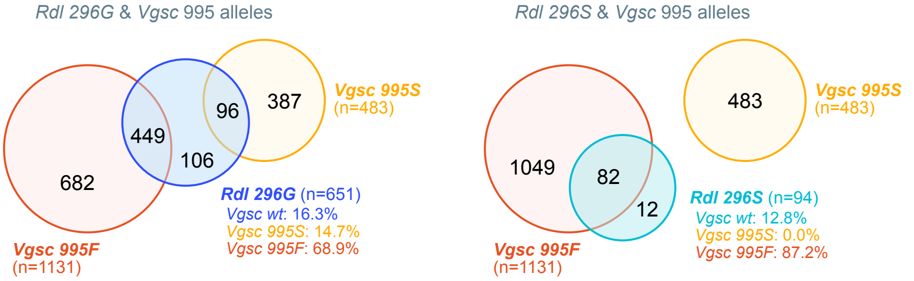

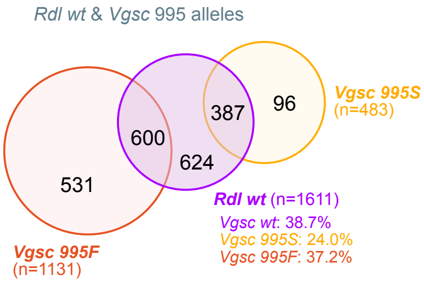

Contingency table: *Rdl* and *Vgsc* alleles

		***Rdl* allele**		
		296G	***296S***	***wt***
***Vgsc* allele**	***995F***	449	82	600
	995S	96	0	387
	wt	106	12	624
	Total	651	94	1611

E) Geographical co-occurrence

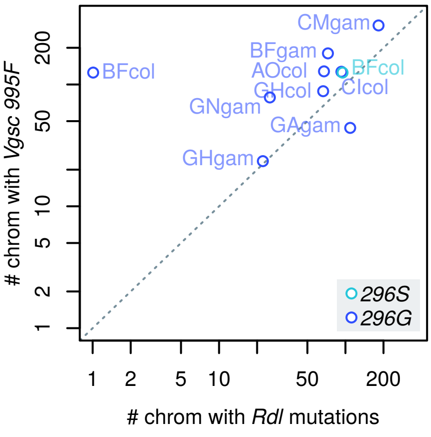

F) Overlap of *Rdl* codon 296 and *Vgsc* codon 995 alleles, per chromosome & populations

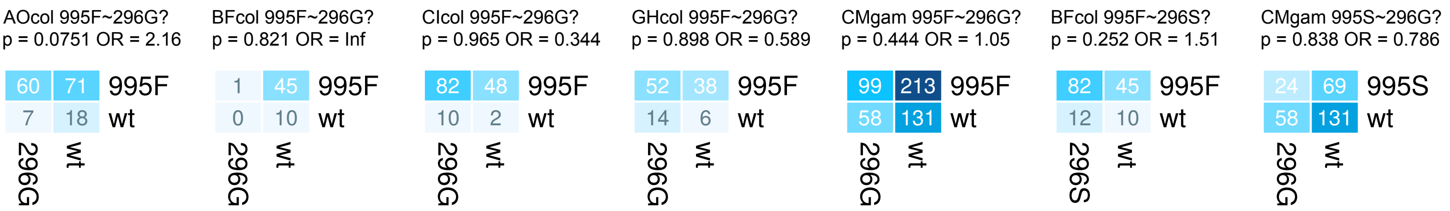

Supplement: msaa128_supplementary_data [file msaa128_supplementary_data.zip › sm8_freqs_vgsc_rd.pdf]
