# Supplementary figures and images for: Evolution of the Insecticide Target Rdl in African Anopheles Is Driven by Interspecific and Interkaryotypic Introgression
Source: Mol Biol Evol. 2020 May 21;37(10):2900–17. doi: 10.1093/molbev/msaa128 (PMC7530614; doi:10.1093/molbev/msaa128)

### A) Alignment of *Rdl* orthologs

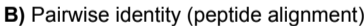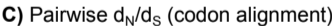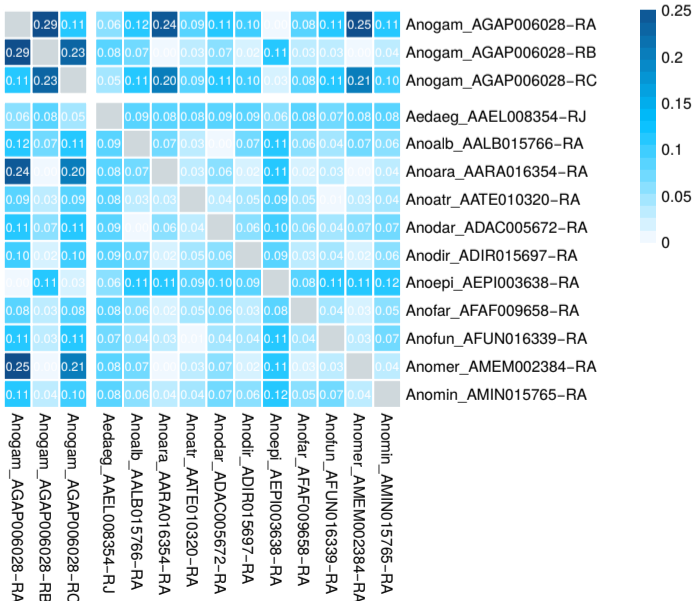

Supplement: msaa128_supplementary_data [file msaa128_supplementary_data.zip › sm4_rdl_mosquito_alignment.pdf]

Supplementary Material 9

A) PCA: PC1 ~ PC2

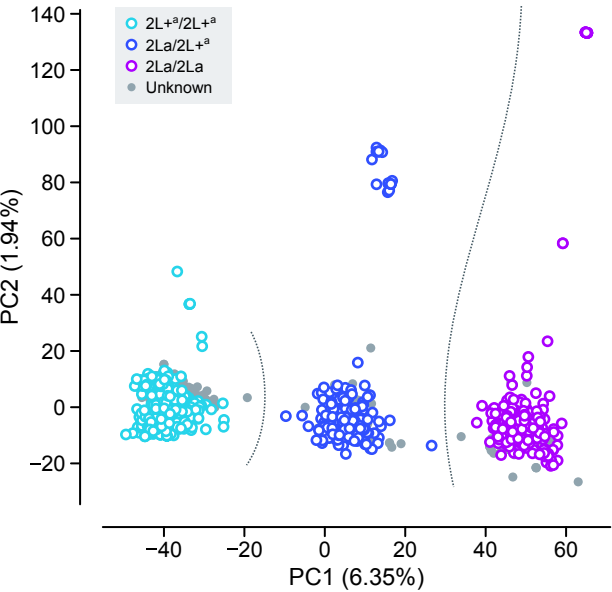

B) PCA: PC1 ~ PC3

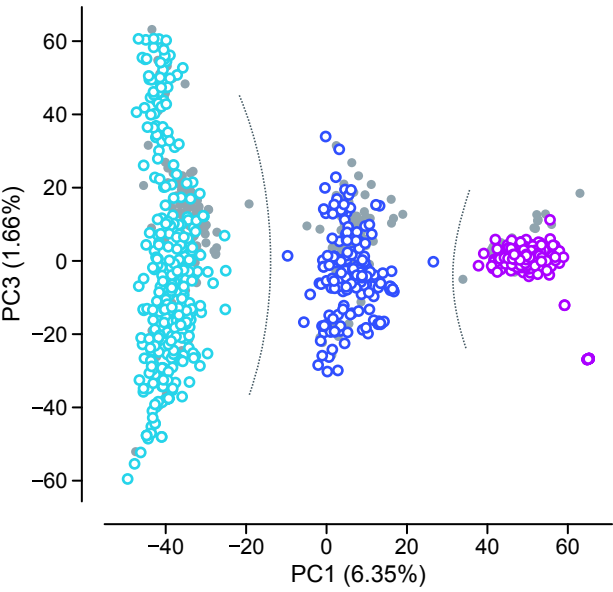

C) Variance explained per component

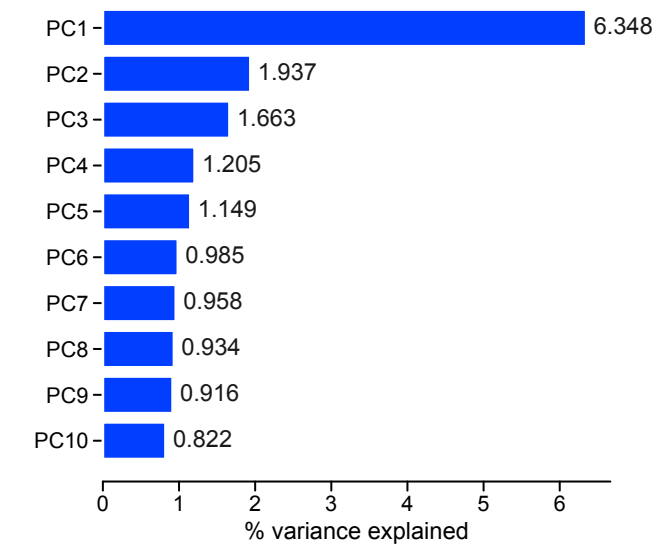

Supplement: msaa128_supplementary_data [file msaa128_supplementary_data.zip › sm9_PCAkaryotype_B.pdf]
